# *Tropheryma whipplei* pneumonia: a retrospective case series of nine patients with treatment response

**DOI:** 10.3389/fmed.2026.1883057

**Published:** 2026-06-29

**Authors:** Qiusheng Yang, Yingjie Chen, Lijuan Chen, Shuo Wei

**Affiliations:** Department of Infectious Diseases, Fuzhou University Affiliated Provincial Hospital, Fujian Medical University, Fuzhou, Fujian, China

**Keywords:** bronchoalveolar lavage fluid, colonization, doxycycline, metagenomic next-generation sequencing, pneumonia, therapeutic response, trimethoprim-sulfamethoxazole, *Tropheryma whipplei*

## Abstract

**Background:**

The detection of *Tropheryma whipplei* in respiratory specimens has increased with metagenomic next-generation sequencing (mNGS), yet distinguishing colonization from active infection remains challenging. In clinical settings lacking quantitative PCR or pathological confirmation, practical approaches to guide treatment decisions are urgently needed.

**Methods:**

We retrospectively analyzed nine patients (January 2019–January 2024) with *T. whipplei* detected by bronchoalveolar lavage fluid (BALF) mNGS. All patients initially received cefoperazone-sulbactam (3.0 g q8h) as empirical therapy for 3–5 days without improvement. Targeted therapy (ceftriaxone or meropenem combined with doxycycline or trimethoprim-sulfamethoxazole) was subsequently initiated. We describe clinical characteristics and treatment outcomes.

**Results:**

Among nine patients (3 male, 6 female; mean age 59 years, range 32–79), six (67%) were immunosuppressed. Primary manifestations included fever (67%), cough with sputum (89%), and dyspnea (78%). Common laboratory findings were anemia (67%), lymphocytopenia (67%), hypoalbuminemia (100%), and elevated inflammatory markers (78%). Chest CT predominantly showed patchy ground-glass opacities. Eight cases (89%) had co-infections. All patients showed no improvement after initial cefoperazone-sulbactam therapy. After targeted therapy was initiated, eight patients (89%) achieved defervescence within 3–5 days, with resolution of pulmonary infiltrates on follow-up CT within 10–14 days. Among these eight responders, one patient (Case 4) underwent repeat BALF mNGS which demonstrated a >99.99% reduction in *T. whipplei* (from 6,100,499 to 383 reads), reported in the suspected colonizer list rather than the pathogen panel. One non-responder (Case 8) showed a >99% reduction in *T. whipplei* read count on repeat BALF mNGS after targeted therapy, but ultimately died of polymicrobial sepsis from multidrug-resistant co-pathogens. No relapse occurred during 1-year follow-up.

**Conclusion:**

This retrospective case series suggests that rapid improvement after adding targeted anti-*T. whipplei* therapy is compatible with possible *T. whipplei*-associated infection in selected mNGS-positive patients, rather than colonization alone. Sequential mNGS showed marked burden reduction in two cases. These observations require prospective validation.

## 1 Introduction

*Tropheryma whipplei*, a Gram-positive actinomycete and the causative agent of classic Whipple’s disease (1), is primarily characterized by weight loss, arthralgia, diarrhea, and abdominal pain, and can be fatal if untreated (2). However, *T. whipplei* may also cause acute infections such as pneumonia, bacteremia, and gastroenteritis (3). In recent years, the widespread application of metagenomic next-generation sequencing (mNGS) technology has significantly increased the detection rate of this bacterium in respiratory specimens (4, 5).

However, a critical clinical dilemma has emerged: does *T. whipplei* detected by mNGS indicate active infection or merely asymptomatic colonization? This bacterium may be present in the saliva of asymptomatic carriers, colonize the respiratory tract of healthy individuals, or cause aspiration pneumonia in combination with oral flora (6). Therefore, the clinical significance of respiratory detection requires clarification.

In a recent multicenter retrospective study, Sun et al. (7) analyzed 91 patients with BALF-mNGS-positive *T. whipplei*. They found that up to 85% of detections represented colonization, while only 15% were confirmed as pneumonia. The study emphasized that mNGS read counts and quantitative PCR (qPCR) validation aid in differentiating infection from colonization. It cautioned clinicians against initiating targeted treatment based solely on mNGS positivity. This finding has important clinical implications for clinical management but poses a new challenge: how to accurately identify patients requiring treatment for *T. whipplei* pneumonia when qPCR or pathological confirmation is unavailable in clinical practice.

We therefore examined nine patients with *T. whipplei* detected by BALF-mNGS. All patients initially received cefoperazone-sulbactam as empirical therapy without improvement; targeted therapy (doxycycline or trimethoprim-sulfamethoxazole) was subsequently initiated. We describe the clinical characteristics and share our observation that rapid improvement in most patients after adding targeted therapy may help identify possible *T. whipplei*-associated infection in selected mNGS-positive patients.

## 2 Materials and methods

### 2.1 Study subjects and inclusion/exclusion criteria

We retrospectively collected patients hospitalized in the Department of Respiratory and Critical Care Medicine, Department of Infectious Diseases, and Department of Critical Care Medicine at our hospital between January 2019 and January 2024.

Inclusion criteria (all criteria must be satisfied concurrently):

(1) Presence of pneumonia-related clinical symptoms (fever, cough, sputum production, dyspnea, etc.);

(2) Chest CT reveals new pulmonary infiltrates (patchy shadows, ground-glass opacities, consolidation, etc.);

(3) *T. whipplei* nucleic acid sequences are detected in BALF by mNGS;

(4) Received targeted anti-*T. whipplei* treatment (doxycycline or trimethoprim-sulfamethoxazole) during hospitalization.

Exclusion criteria (meeting any of the following):

(1) Absence of pneumonia-related clinical manifestations, or pulmonary lesions being chronic/non-infectious;

(2) Only mNGS positivity without clinical suspicion of pathogenicity, and no targeted treatment administered;

(3) More plausible causative agents were identified during follow-up.

### 2.2 Treatment description

Given that our institution does not routinely perform *T. whipplei* qPCR or lung biopsy PAS staining, this study documented a treatment-response pattern for infection inference. All patients initially received cefoperazone-sulbactam (3.0 g q8h) for 3–5 days based on clinical manifestations and imaging findings. This antibiotic was selected based on the following rationale: Given that all patients in this cohort were immunosuppressed or had received antibiotics within the past 3 months, cefoperazone-sulbactam, as a β-lactam/β-lactamase inhibitor combination, served as the preferred empirical treatment for pneumonia in these patients, covering common nosocomial pathogens. This drug exhibits poor penetration against the obligate intracellular bacterium *T. whipplei* due to its inability to effectively reach intracellular penicillin-binding proteins (PBPs) involved in cell-wall synthesis (8). As an obligate intracellular bacterium that replicates within macrophage vacuoles and lacks key biosynthetic pathways, *T. whipplei* is dependent on host-derived substrates and maintains an intimate interaction with its host (8), rendering beta-lactam antibiotics ineffective. During this period, clinical symptoms (including temperature, cough, dyspnea, and oxygenation index) and imaging changes were recorded daily.

**FIGURE 1:**
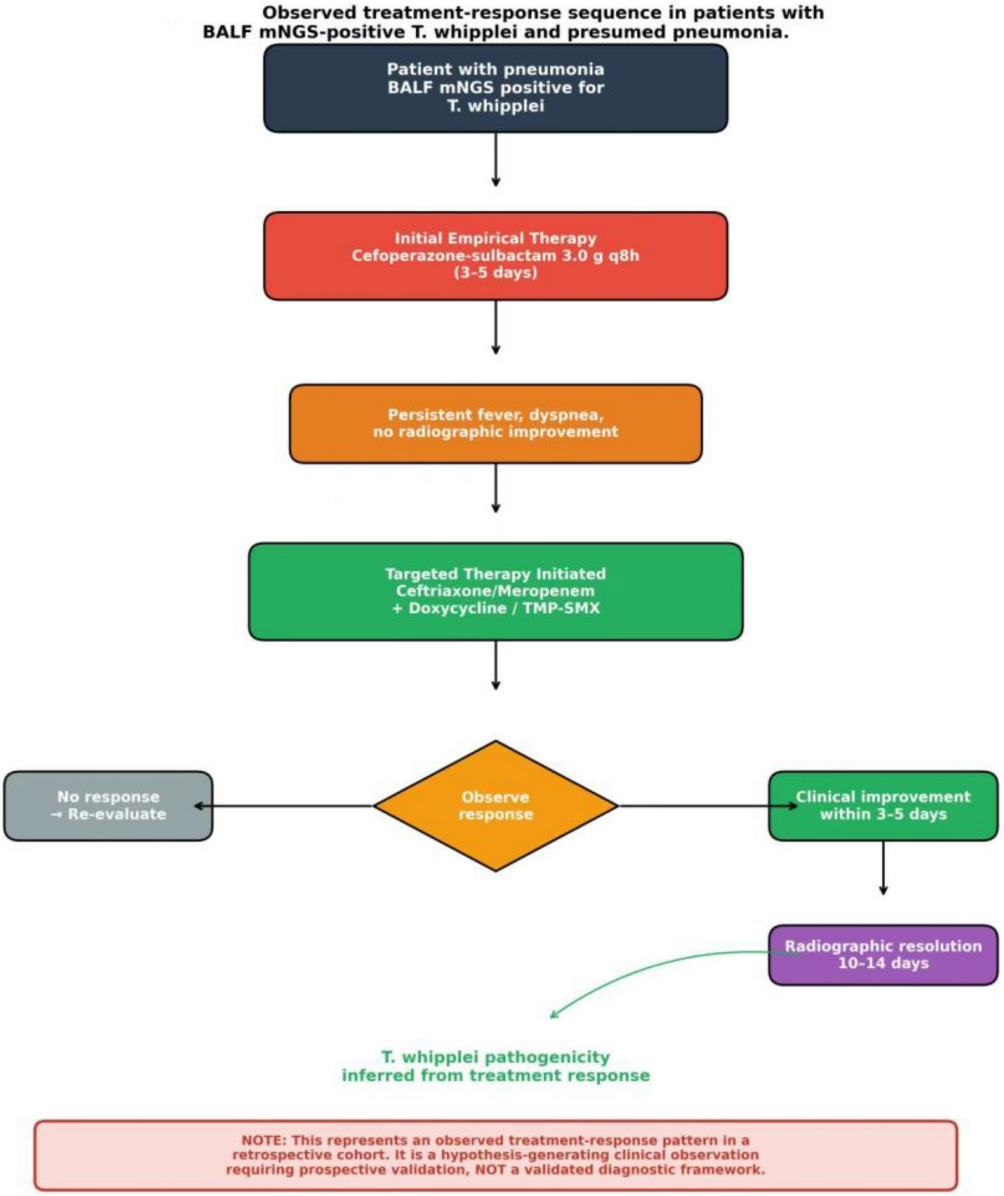
Observed treatment-response sequence in patients with BALF mNGS-positive *T. whipplei*.

After BALF-mNGS reported *T. whipplei* detection, targeted therapy was initiated: ceftriaxone (2 g qd) combined with doxycycline (100 mg q12h), ceftriaxone (2 g qd) combined with trimethoprim-sulfamethoxazole (960 mg q12h), meropenem (1 g q8h) combined with doxycycline (100 mg q12h), or meropenem (1 g q8h) combined with trimethoprim-sulfamethoxazole (960 mg q12h). Ceftriaxone and meropenem were selected as the induction component based on established guidelines for classic Whipple’s disease, where they represent the standard initial therapy for 2 weeks (8). Doxycycline offers favorable intracellular penetration (8, 9). Trimethoprim-sulfamethoxazole provides reliable anti-*T. whipplei* activity through alternative mechanisms (8, 9). Both agents serve as the maintenance backbone for long-term eradication. In this acute pneumonia setting, we combined the induction and maintenance agents simultaneously to achieve rapid control of both co-infecting pathogens and intracellular *T. whipplei*. Ceftriaxone and meropenem also provide broad-spectrum coverage for co-infecting pathogens, which were present in 89% of cases. Figure 1 illustrates the observed treatment sequence.

### 2.3 Data collection

We collected patient clinical data via the electronic medical record system, including:

- Demographic information: age and gender;
- Underlying diseases and immune status: immunocompromised hosts were defined according to the ‘Expert Consensus on Diagnosis and Treatment of Pneumonia in Immunocompromised Hosts’ (10)
- Clinical manifestations: fever, cough, sputum production, dyspnea, chest pain, and arthralgia;
- Laboratory tests: complete blood count, C-reactive protein (CRP), erythrocyte sedimentation rate (ESR), procalcitonin (PCT), albumin, D-dimer, and CD4+ T lymphocyte count;
- Imaging examination: Chest CT findings;
- Microbiological examination: BALF-mNGS results, conventional smear and culture results;
- Treatment and outcomes: Antibiotic treatment regimen, specific timeline, daily temperature records, dyspnea score, oxygenation index changes, CT re-examination results, and prognosis.

### 2.4 BALF mNGS testing

All patients underwent BALF collection from lesion sites via flexible bronchoscopy guided by chest CT localization. DNA extraction, library preparation and metagenomic sequencing on the Illumina NovaSeq platform (minimum 20 million reads per sample) followed standard operating procedures. Quality control included: (1) removal of low-quality reads (Q-score < 30); (2) removal of adapter sequences; (3) removal of human-derived sequences by alignment to the human reference genome (hg19) using Burrows-Wheeler Alignment (5); (4) pathogen identification requiring non-human reads >50, coverage >3 unique reads mapped to the species level, and species-level match in the NCBI RefSeq microbial database (5). Contamination prevention was ensured through negative control blanks in each sequencing batch. Negative control blanks in each sequencing batch showed no *T. whipplei* detection, supporting that the reported sequences were not derived from environmental or reagent contamination.

### 2.5 Statistical analysis

Given the small sample size (*n* = 9) and the descriptive nature of this study, no inferential statistical tests were performed. Data analysis employed descriptive statistical methods only. Continuous variables are presented as median (range) or mean ± standard deviation, while categorical variables are expressed as number of cases (percentage).

## 3 Results

### 3.1 Clinical characteristics and treatment response

Among nine evaluated patients with *T. whipplei* pneumonia, three were male and six were female, with a mean age of 59 years (range 32–79 years). Seven cases (78%) had acute onset (duration: 1–10 days), one had chronic onset (>2 months), and one was incidentally discovered during physical examination. Six patients (67%) were immunosuppressed, with underlying diseases including diabetes (2 cases), malignancy (2 cases), rheumatoid disease (4 cases), and chronic kidney disease (2 cases). The median hospital stay was 14 days (range: 10–28 days).

Therapeutic Response:

All patients received cefoperazone-sulbactam (3.0 g q8h) for 3–5 days (median 4 days). During this period, all patients had persistent fever (body temperature 37.8–39.5 °C), unresolved dyspnea (respiratory rate 28–35 breaths/min), and no improvement in oxygenation index (PaO_2_/FiO_2_). Chest CT re-examinations at 3–5 days showed no resolution or even progression of pulmonary infiltrates.

After mNGS detection of *T. whipplei*, targeted therapy was initiated : ceftriaxone plus doxycycline (Cases 1–3, 6), ceftriaxone plus trimethoprim-sulfamethoxazole (Cases 7, 9), meropenem plus doxycycline (Case 4), and meropenem plus trimethoprim-sulfamethoxazole (Case 5). Within 3–5 days of targeted therapy initiation, eight patients (89%) achieved normal body temperature, demonstrated significant relief of dyspnea, and showed improved oxygenation index. Follow-up chest CT scans at 10–14 days showed heterogeneous improvement in pulmonary infiltrates, with partial resolution in most cases. Case 8 died of polymicrobial sepsis before repeat imaging could be performed. Radiographic outcomes were heterogeneous. Several cases showed partial improvement, while others had stable or slowly resolving infiltrates. Imaging assessment remained descriptive without standardized scoring or blinded review.

Case 4 also underwent two BALF mNGS tests. The first test on admission detected *T. whipplei* (6,100,499 reads) along with *Acinetobacter baumannii*, *Corynebacterium striatum*, and *Pseudomonas aeruginosa*. After approximately 10 days of treatment… the second BALF mNGS showed that *T. whipplei* had decreased to 383 reads (>99.99% reduction), reported in the suspected colonizer list rather than the pathogen panel, while other pathogens (*Enterococcus faecium*, *Ralstonia mannitolilytica*, *Corynebacterium striatum*, *Pseudomonas aeruginosa*) were identified.

One patient (Case 8) had two BALF mNGS tests. He received cefoperazone-sulbactam initially. After 3 days, the first BALF mNGS returned 15,092 *T. whipplei* sequences, and targeted therapy was initiated with meropenem plus doxycycline. About 1 week later, the second BALF mNGS showed the count had dropped to 18—a decline of more than 99%. This striking reduction in sequence count is the most compelling objective finding in our cohort, indicating that the *T. whipplei* burden dropped markedly after targeted therapy. The second test also detected *Enterococcus faecium* and *Pseudomonas aeruginosa*, indicating mixed infection, so linezolid was added. Bronchial secretions grew multidrug-resistant *Acinetobacter baumannii*, blood cultures grew multidrug-resistant *Klebsiella pneumoniae* and *Enterococcus faecium*, and urine culture grew *Candida parapsilosis*. Roughly 50 days after the second mNGS, the patient died of polymicrobial sepsis caused by these multidrug-resistant organisms, complicated by septic shock and acute renal failure.

During the 1-year follow-up, patients were evaluated at outpatient clinics at 1, 3, 6, and 12 months post-discharge, including clinical symptom assessment and chest CT when indicated. No patient received long-term suppressive anti-*T. whipplei* therapy after discharge, and none experienced recurrence of *T. whipplei* pneumonia.

The primary clinical manifestations included fever (6/9, 67%), cough and sputum production (8/9, 89%), and dyspnea (7/9, 78%), with only one case (11%) presenting with joint pain. No patients exhibited classic manifestations of Whipple’s disease such as weight loss, diarrhea, or abdominal pain (Table 1).

**TABLE 1:** Demographic, clinical characteristics, laboratory findings, and treatment outcomes of 9 patients with clinically evaluated *Tropheryma whipplei* pneumonia.

Parameter	Case 1	Case 2	Case 3	Case 4	Case 5	Case 6	Case 7	Case 8	Case 9
Age/years	59	36	64	32	71	79	68	65	57
Sex	Male	Male	Female	Female	Female	Male	Female	Female	Female
Underlying conditions									
Diabetes mellitus	Yes	No	Yes	No	No	No	No	No	No
Malignancy	Yes	No	No	No	No	Yes	No	No	No
Rheumatoid disease	No	No	Yes	No	Yes	No	Yes	No	Yes
Chronic kidney disease	Yes	No	No	Yes	No	No	No	No	No
Other immunocompromising conditions	Mesangial proliferative glomerulonephritis, malignant mesothelioma, disseminated cryptococcosis	Hepatitis B carrier	Polymyositis with interstitial pneumonia	Right renal transplant	Dermatomyositis (anti-SAE), lower extremity venous thrombosis	Prostate cancer with bone metastases	Rheumatoid arthritis	Depression, hypertension, cerebral infarction, pulmonary embolism	Undifferentiated connective tissue disease
Clinical manifestations									
Fever	Yes	Yes	No	Yes	No	Yes	Yes	Yes	Yes
Cough with sputum	Yes	Yes	Yes	Yes	No	Yes	Yes	Yes	No
Dyspnea	Yes	Yes	Yes	Yes	No	Yes	Yes	Yes	Yes
Chest pain	No	No	Yes	Yes	No	No	No	No	Yes
Arthralgia	No	No	No	No	No	No	No	No	Yes
Laboratory findings									
WBC (×10^9^/L)	13.4	11.1	5.4	19.9	4.2	6.3	5.6	6.6	5.8
Neutrophils (×10^9^/L)	12.8	8.5	2.7	19.4	2.9	4.4	2.3	4.6	4.6
Lymphocytes (×10^9^/L)	0.3	0.9	2.0	0.1	0.9	1.1	2.5	1.5	0.8
Hemoglobin (g/L)	100	127	130	61	108	113	86	76	131
Albumin (g/L)	23	35	35	30	38	35	32	31	32
Platelets (×10^9^/L)	339	206	228	91	142	426	162	123	224
CRP (mg/L)	147	69	4.6	358	13.5	64	16	264	71
ESR (mm/h)	106	37	31	–	27	75	63	>140	74
PCT (ng/mL)	0.25	0.25	0.25	>100	0.25	0.25	0.15	9.1	0.25
D-dimer (mg/L)	2.4	0.23	0.61	6.7	0.86	0.75	1.38	4.6	0.3
CD4+ T cells (cells/μL)	83	981	746	27	298	415	1307	396	1236

WBC, white blood cell count; CRP, C-reactive protein; ESR, erythrocyte sedimentation rate; PCT, procalcitonin.

### 3.2 Laboratory tests

Laboratory test results are presented in Table 1. Common abnormalities included anemia (6/9, 67%), decreased lymphocyte count (6/9, 67%), hypoalbuminemia (9/9, 100%), elevated D-dimer (7/9, 78%), elevated CRP (7/9, 78%), and elevated erythrocyte sedimentation rate (7/9, 78%). Procalcitonin (PCT) elevation was observed in only two cases (22%). Four patients (44%) had decreased CD4+ T-lymphocyte counts.

### 3.3 Chest CT findings

**FIGURE 2:**
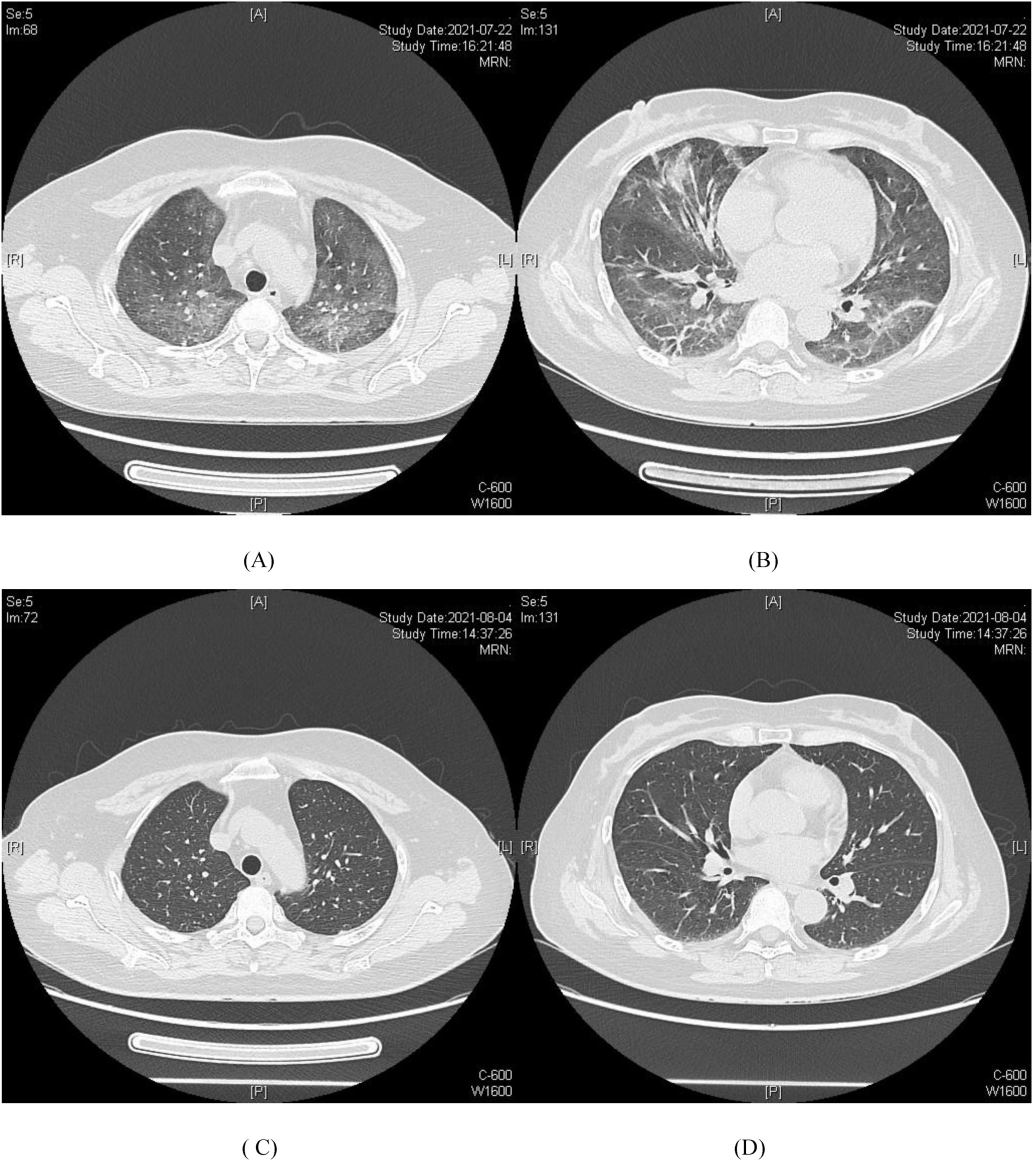
Representative chest CT images of Case 9 before **(A,B)** and 9 days after **(C,D)** targeted therapy, showing marked improvement in bilateral patchy infiltrates.

Chest CT manifestations were heterogeneous, primarily characterized by unilateral or bilateral, patchy or diffuse consolidation or ground-glass opacities (GGO). Some cases exhibited air bronchograms and were accompanied by unilateral or bilateral pleural effusions. Representative chest CT images of Case 9 before and 9 days after targeted therapy are shown in Figure 2. In this patient, *Pneumocystis jirovecii* was co-detected with only 7 reads; however, given the preserved CD4+ T-cell count (1,236 cells/μL) and negative BALF microscopy, this was considered colonization rather than active Pneumocystis pneumonia, although the contribution of TMP-SMX to Pneumocystis suppression cannot be fully excluded. Radiographic assessment in this case series was descriptive, without blinded review, standardized scoring, or a formal imaging endpoint.

### 3.4 Microbiological findings

*Tropheryma whipplei* was detected by BALF-mNGS in all nine patients. Only one case represented a single infection, while the remaining eight cases (89%) showed co-infection. Common co-pathogens included *Pneumocystis jirovecii* (2 cases), Epstein-Barr virus (4 cases), *Corynebacterium striatum* (2 cases), and CMV (2 cases), with additional high-read detection of *Moraxella catarrhalis*, *Acinetobacter baumannii*, and *Pseudomonas aeruginosa* in individual cases (Table 2). Conventional bacterial and fungal cultures of initial BALF were negative in all patients.

**TABLE 2:** Chest CT findings, microbiological results, subsequent targeted therapy, and outcomes of 9 patients with clinically evaluated *Tropheryma whipplei* pneumonia.

Case	Onset	Chest CT findings	mNGS results (*T. whipplei* read count)	Subsequent targeted therapy*	Radio-graphic outcome	Outcome
1	1 day	Bilateral patchy, nodular infiltrates with ground-glass opacity, partial cavitation, bilateral pleural effusion	*T. whipplei* (4,985,876), *Streptococcus constellatus* (22,184), *Cryptococcus neoformans* (263,028), *Pneumocystis jirovecii* (373), HHV-7 (142), CMV (90), EBV (78), Torque teno virus 16 (23)	Ceftriaxone + Doxycycline	Improved	Discharged
2	7 days	Right middle and lower lobe patchy infiltrates with air bronchograms, tree-in-bud signs	*T. whipplei* (593), *Mycoplasma pneumoniae* (52,592), *Candida albicans* (201), EBV (399)	Ceftriaxone + Doxycycline	Improved	Discharged
3	2 months	Bilateral patchy, linear infiltrates with ground-glass opacity and consolidation, predominantly lower lobes	*T. whipplei* (178), *Streptococcus oralis* (1)	Ceftriaxone + Doxycycline	Improved	Discharged
4	1 day	Bilateral multiple patchy infiltrates with consolidation, predominantly right lung, air bronchograms, right pleural effusion	*T. whipplei* (6,100,499), *Acinetobacter baumannii* (308,705), *Corynebacterium striatum* (147,191), *Pseudomonas aeruginosa* (96,643) Second BALF mNGS: *T. whipplei* (383), *Enterococcus faecium* (1,073,445), *Ralstonia mannitolilytica* (524,073), *Corynebacterium striatum* (10,787), *Pseudomonas aeruginosa* (1,137)	Meropenem + Doxycycline	Improved	Discharged‡
5	Unknown	Bilateral multiple patchy infiltrates, subpleural distribution, blurred margins, short spicules, pleural thickening	*T. whipplei* (947), *Haemophilus influenzae* (4), *Mycobacterium chelonae* (3), *Neisseria subflava* (1,259), *Streptococcus mitis* (444), *Rothia mucilaginosa* (539), *Prevotella polymorpha* (182)	Meropenem + Compound sulfamethoxazole	Improved	Discharged
6	10 days	Bilateral multiple large patchy and consolidative infiltrates, blurred margins, left pleural effusion	*T. whipplei* (10,970), *Moraxella catarrhalis* (3,272,192), *Streptococcus constellatus* (38,864), EBV (23,901), CMV (8,315), *Streptococcus pneumoniae* (1,414)	Ceftriaxone + Doxycycline	Improved	Discharged
7	10 days	Bilateral diffuse patchy ground-glass infiltrates, blurred margins, predominantly upper lobes	*T. whipplei* (1,206)	Ceftriaxone + Compound sulfamethoxazole	Improved	Discharged
8	7 days	Bilateral multiple patchy, fluffy, ground-glass infiltrates, blurred margins, partial consolidation, air bronchograms, bilateral pleural effusion	*T. whipplei* (15,092), EBV (5), Multidrug-resistant *Corynebacterium* (62), *Mycobacterium chelonae* (3) Second BALF mNGS: *T. whipplei* (18),*Enterococcus faecium* (54), *Pseudomonas aeruginosa* (10)	Meropenem + Doxycycline	Death before repeat CT	Death † (polymicrobial sepsis). Cultures: Bronchial secretion: MDR *Acinetobacter baumannii*; Blood: MDR *Klebsiella pneumoniae*, *Enterococcus faecium*; Urine: *Candida parapsilosis*.
9	7 days	Bilateral diffuse multiple patchy, linear infiltrates, blurred margins, partial ground-glass opacity	*T. whipplei* (290), *Pneumocystis jirovecii* (7)	Ceftriaxone + Compound sulfamethoxazole	Improved (see Figure 2)	Discharged

*Subsequent Targeted Therapy was initiated after mNGS results confirmed *T. whipplei* detection. ‡ Case 4 had two BALF mNGS tests approximately 10 days apart. The second test showed *T. whipplei* 383 reads (>99.99% reduction), reported in the suspected colonizer list rather than the pathogen panel.† Case 8 had two BALF mNGS tests approximately 8 days apart. The second test showed *T. whipplei* read count declined from 15,092 to 18 (>99% reduction), indicating the *T. whipplei* burden dropped markedly after targeted therapy. Death was due to polymicrobial sepsis from multidrug-resistant *Acinetobacter baumannii*, *Klebsiella pneumoniae*, and *Enterococcus faecium*.

The mNGS read counts varied considerably across this patient cohort (range: 178 to 6,100,499). Two patients (Cases 4 and 8) underwent sequential BALF mNGS testing. In Case 4, *T. whipplei* decreased from 6,100,499 to 383 reads (>99.99% reduction) on the second test after approximately 10 days of targeted therapy; the laboratory algorithm classified this as a suspected colonizer rather than a pathogen, further supporting effective suppression of the pathogen burden. In Case 8, the read count declined from 15,092 to 18 (>99% reduction) after 8 days. Most cases showed high read counts consistent with active infection, while some cases (e.g., Case 3 with 178 reads) had relatively low read counts. Detailed mNGS reports are provided in the Supplementary materials. This variability aligns with the study by Sun et al. (7), which demonstrated significant overlap in read counts between the pneumonia and colonization groups, emphasizing that read counts alone cannot reliably differentiate infection from colonization. In this study, pathogenic inference was primarily based on therapeutic response rather than absolute read counts.

### 3.5 Treatment timeline and response (core outcomes)

**FIGURE 3:**
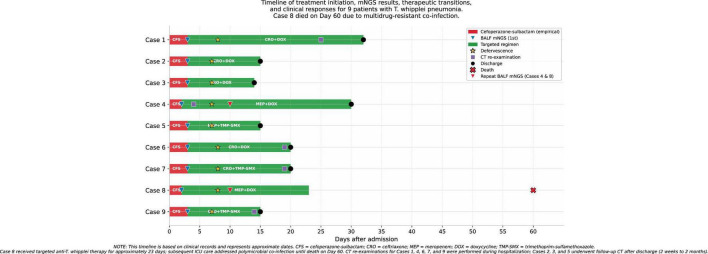
Treatment timeline for 9 patients with *T. whipplei* pneumonia.

Figure 3 shows the treatment timeline for all nine patients.

All patients received cefoperazone-sulbactam (3.0 g q8h) for 3–5 days as initial empirical therapy before the subsequent targeted regimens listed in Table 2 were initiated.

## 4 Discussion

### 4.1 Redefining the identification of *T. whipplei* pneumonia in the mNGS era

*Tropheryma whipplei* is frequently detected by mNGS in respiratory specimens, yet most detections represent colonization. Sun et al. (7) confirmed pneumonia in only 14 of 91 patients (15%) using qPCR, but most hospitals lack this capability. Our nine patients do not establish a diagnostic rule; they document a simple clinical observation: patients who fail cefoperazone-sulbactam yet improve after adding doxycycline or TMP-SMX may have possible *T. whipplei*-associated infection in selected mNGS-positive patients.

The core observation of this study is that most patients with possible *T. whipplei* pneumonia failed to improve on cefoperazone-sulbactam empirical therapy, yet responded rapidly after targeted therapy was initiated. *T. whipplei* is an obligate intracellular bacterium that replicates within macrophage vacuoles and lacks key biosynthetic pathways, making it dependent on host-derived substrates and maintaining an intimate interaction with its host (8). Because of this intracellular niche, beta-lactam antibiotics such as cefoperazone-sulbactam achieve poor intracellular concentrations and cannot effectively reach penicillin-binding proteins (PBPs) involved in cell-wall synthesis within macrophages (8, 11).

However, we acknowledge that cefoperazone-sulbactam is not a true experimental control; it is an active broad-spectrum antibiotic, and failure to improve within 3–5 days does not prove *T. whipplei* pathogenicity. The treatment response pattern we observed is a clinical heuristic, not a validated diagnostic protocol. Previous reports have similarly documented rapid clinical and radiographic improvement after targeted therapy. Zhang et al. (11) reported a *T. whipplei* pulmonary cavity that resolved completely within 6 weeks after ceftriaxone and TMP-SMX treatment, and Fang et al. (12) described clinical improvement in *T. whipplei* pneumonia after doxycycline-based therapy.

Repeat BALF mNGS in two patients supports this observation. In Case 8, despite ultimately succumbing to polymicrobial sepsis from multidrug-resistant co-pathogens, the patient showed a >99% reduction in *T. whipplei* mNGS read count (from 15,092 to 18) after initiating targeted therapy with meropenem plus doxycycline, indicating that the targeted regimen was associated with a marked reduction in *T. whipplei* burden; however, this reduction alone did not prevent death from persistent polymicrobial sepsis. Similarly, Case 4 demonstrated near-complete clearance of *T. whipplei* on repeat BALF mNGS with the pathogen burden decreasing from 6,100,499 to 383 reads, reported in the suspected colonizer list rather than the pathogen panel, while other co-pathogens persisted. These sequential mNGS findings are compatible with targeted anti-*T. whipplei* activity, but do not establish causality.

### 4.2 Treatment response and co-infection confounding

We distinguish active *T. whipplei* pulmonary infection (causing symptoms and radiographic abnormalities) from asymptomatic colonization, transient oral carriage, and aspiration-related detection during bronchoscopy. The distinction between colonization and infection in *T. whipplei*-positive respiratory specimens remains challenging. Lagier et al. (6) emphasized that *T. whipplei* DNA in BALF may represent either colonization or active infection, and Lin et al. (5) noted that infection, colonization, and prognosis of *T. whipplei* in the lung still need to be studied. This study aimed to identify active *T. whipplei* pulmonary infection, though we acknowledge that mNGS alone cannot definitively establish tissue invasion without histopathological confirmation.

We adopted initial broad-spectrum coverage followed by targeted therapy. Clinical improvement after adding targeted therapy may reflect multiple factors: treatment of co-pathogens, natural disease course, or the targeted agent itself. The confounding effect of co-infections is a recognized limitation across the field. Lin et al. (5) found that only 21.4% of mNGS-positive *T. whipplei* patients were ultimately diagnosed with pneumonia, with 34.3% having tuberculosis and 21.4% lung tumors, underscoring the complexity of attributing clinical improvement to a single pathogen.

Case 8 exemplifies this confounding: although *T. whipplei* read counts declined by >99% after targeted therapy, the patient ultimately died of polymicrobial sepsis caused by multidrug-resistant *Acinetobacter baumannii*, *Klebsiella pneumoniae*, and *Enterococcus faecium*. This indicates that suppressing *T. whipplei* alone is insufficient when severe co-infections persist, and clinical outcomes depend on the overall microbial landscape.

### 4.3 Specificity of anti-*T. whipplei* therapy

Case 4 illustrates this point clearly: after approximately 10 days repeat BALF mNGS showed near-complete clearance of *T. whipplei* (decreased from 6,100,499 to 383 reads, reported in the suspected colonizer list rather than the pathogen panel), yet other pathogens (*Enterococcus faecium*, *Pseudomonas aeruginosa*, *cytomegalovirus*) persisted or newly appeared. The fact that *T. whipplei* was eliminated while other microbes remained is consistent with targeted anti-*T. whipplei* activity, though we cannot exclude non-specific effects or the contribution of other therapeutic components.

In Case 9, BALF-mNGS detected *Pneumocystis jirovecii* (7 reads) alongside *T. whipplei* (290 reads). The patient had undifferentiated connective tissue disease and was receiving methylprednisolone 16 mg QD. However, her CD4+ T-cell count was 1,236 cells/μL, well above the non-HIV Pneumocystis pneumonia risk threshold of <200 cells/μL (13). Long et al. (14) reported *Pneumocystis jirovecii* colonization in 17.14% of non-AIDS immunosuppressed patients. Given low Pneumocystis reads, preserved CD4 count, and negative BALF microscopy, this was considered colonization rather than active Pneumocystis pneumonia. The observed radiographic improvement was therefore more consistent with response to anti-*T. whipplei* therapy than with anti-Pneumocystis treatment; however, the contribution of TMP-SMX to Pneumocystis suppression cannot be fully excluded.

### 4.4 Pharmacological rationale for targeted therapy

The treatment response pattern was consistent across all patients who responded: they failed to improve on cefoperazone-sulbactam, but improved after targeted therapy was initiated. Doxycycline, with its lipophilic nature, crosses cell membranes and reaches therapeutic concentrations inside macrophages where *T. whipplei* resides (6, 7). TMP-SMX provides reliable anti-*T. whipplei* activity: although *T. whipplei* is naturally resistant to trimethoprim due to the absence of dihydrofolate reductase, the sulfamethoxazole component retains activity by inhibiting dihydropteroate synthetase, and the clinical efficacy of the combination regimen has been established in a randomized controlled trial (15).

Ceftriaxone and meropenem were included in the targeted regimens based on established guidelines for classic Whipple’s disease, where they represent the standard initial induction therapy for 2 weeks (8, 15). However, in the context of pulmonary infection, *T. whipplei* primarily resides within alveolar macrophages, and beta-lactam antibiotics achieve poor intracellular concentrations (8, 11). The key therapeutic addition for intracellular eradication was doxycycline or TMP-SMX. The fact that patients did not improve during initial empirical therapy but improved after targeted therapy (including doxycycline or TMP-SMX) was initiated supports the attribution of clinical response to these intracellularly active agents.

Patients did not improve until doxycycline or TMP-SMX was added, which is compatible with a targeted effect on intracellular *T. whipplei*, though we cannot rule out confounding by co-pathogen treatment or natural disease course.

Sequential BALF mNGS in two patients provides preliminary supportive data for this interpretation: repeat BALF mNGS is compatible with the activity of doxycycline (Cases 4 and 8), though it does not prove clinical improvement was caused by this agent alone.

### 4.5 Summary of clinical features

We confirmed that evaluated *T. whipplei* pneumonia primarily occurred in immunocompromised patients (67%), and all severe cases were exclusively observed in this population, aligning with the opportunistic nature of *T. whipplei* as a pathogen. None of the patients in this cohort presented with the classic triad of Whipple’s disease (diarrhea, weight loss, and arthralgia), suggesting that pulmonary infection may be the sole or initial manifestation of *T. whipplei* infection. This finding is in line with literature reports (12, 16, 17).

Laboratory tests revealed that patients with *T. whipplei* pneumonia frequently present with lymphopenia, hypoalbuminemia, low procalcitonin (PCT), and low-level abnormalities in C-reactive protein (CRP)/erythrocyte sedimentation rate (ESR) (mildly elevated). This observation corresponds to the ‘mild inflammatory response’ characteristic documented in the literature (17).

### 4.6 Treatment strategy selection

Regarding co-infections identified in 89% of cases, we adopted a stratified therapeutic approach: initial broad-spectrum coverage followed by targeted therapy. For *T. whipplei*, we infer its pathogenic role through the principle of no improvement with ineffective medication followed by significant improvement with effective medication.

Currently, there is no standardized treatment protocol for *T. whipplei* pneumonia. The standard treatment regimen for classic Whipple’s disease involves initial therapy with ceftriaxone or meropenem for 2 weeks, followed by maintenance therapy with trimethoprim-sulfamethoxazole or doxycycline plus hydroxychloroquine for 1 year (11). In this study, we selected a ceftriaxone/meropenem-based regimen combined with doxycycline or TMP-SMX for acute pneumonia management, rather than the currently internationally recommended doxycycline plus hydroxychloroquine maintenance regimen for classic Whipple’s disease, based on the following considerations:

1. Different treatment goals: All patients in this cohort had acute pneumonia, with the treatment objective being rapid control of lung infection rather than long-term maintenance. Ceftriaxone or meropenem as potent bactericidal agents; combined with either doxycycline (which has good intracellular penetration) or trimethoprim-sulfamethoxazole (with broad-spectrum coverage), can rapidly reduce pathogen load.

2. Hospitalization duration constraint: Given that the median hospital stay was 14 days (range: 10–28) in this study, most patients could not complete the full 2-week induction therapy.

3. Safety considerations: Considering the potential retinal toxicity and drug interactions of hydroxychloroquine, we selected doxycycline or trimethoprim-sulfamethoxazole due to their higher safety profiles during short-term treatment.

4. Therapeutic validation: All patients achieved favorable short-term therapeutic outcomes with no recurrence during 1-year follow-up, indicating the regimen’s efficacy in specific clinical scenarios. For recurrent or refractory cases, however, long-term therapy combining doxycycline and hydroxychloroquine remains recommended.

### 4.7 Limitations

This study has several limitations. Nine patients from a single center constitute a small sample, and the retrospective design introduces selection bias. We included consecutive patients diagnosed with *T. whipplei* pneumonia by BALF-mNGS who received targeted anti-*T. whipplei* therapy during hospitalization; those with incomplete records were excluded. Without a control group of mNGS-positive patients who did not receive targeted therapy, we cannot calculate sensitivity, specificity, or attributable treatment effect. We also lacked qPCR or pathological confirmation, so our pathogenic inference rests on clinical response rather than gold-standard verification. The decision to add doxycycline or TMP-SMX was made by individual clinicians based on mNGS results and clinical judgment, without a standardized protocol. These limitations are consistent with the current state of the field. Lagier et al. (6) performed a case-control study of 1438 BALF samples to address this issue, while Lin et al. (5) analyzed 1725 BALF samples and still concluded that “infection, colonization, and prognosis of *T. whipplei* in the lung still need to be studied.” Given these limitations, this study is intended to generate hypotheses for future prospective validation rather than to establish definitive treatment protocols.

Sequential mNGS data from only two patients are suggestive but insufficient to prove causality. Read counts can be influenced by multiple technical and biological factors, and absence of detection does not equate to microbiological eradication.

## 5 Conclusion

This retrospective case series suggests that rapid improvement in most patients after adding targeted anti-*T. whipplei* therapy is compatible with possible *T. whipplei*-associated infection in selected mNGS-positive patients rather than colonization alone. Sequential BALF mNGS in two patients demonstrated marked reduction or clearance of *T. whipplei* after targeted therapy, providing preliminary observational data consistent with this clinical heuristic. However, these observations require confirmation in larger prospective studies with qPCR or pathological validation.

## Data Availability

The datasets presented in this article are not readily available because of patient privacy and ethical restrictions. Requests to access the datasets should be directed to the corresponding author.
