# Supplementary material for: Tropheryma whipplei pneumonia: a retrospective case series of nine patients with treatment response
Source: Front Med (Lausanne). 2026 Jun 29;13:1883057. doi: 10.3389/fmed.2026.1883057 (PMC13357807; doi:10.3389/fmed.2026.1883057)
Supplement: Supplementary file 1 [file Data_Sheet_1.PDF]

MAPMI™ 超广谱病原微生物 mNGS 检测报告

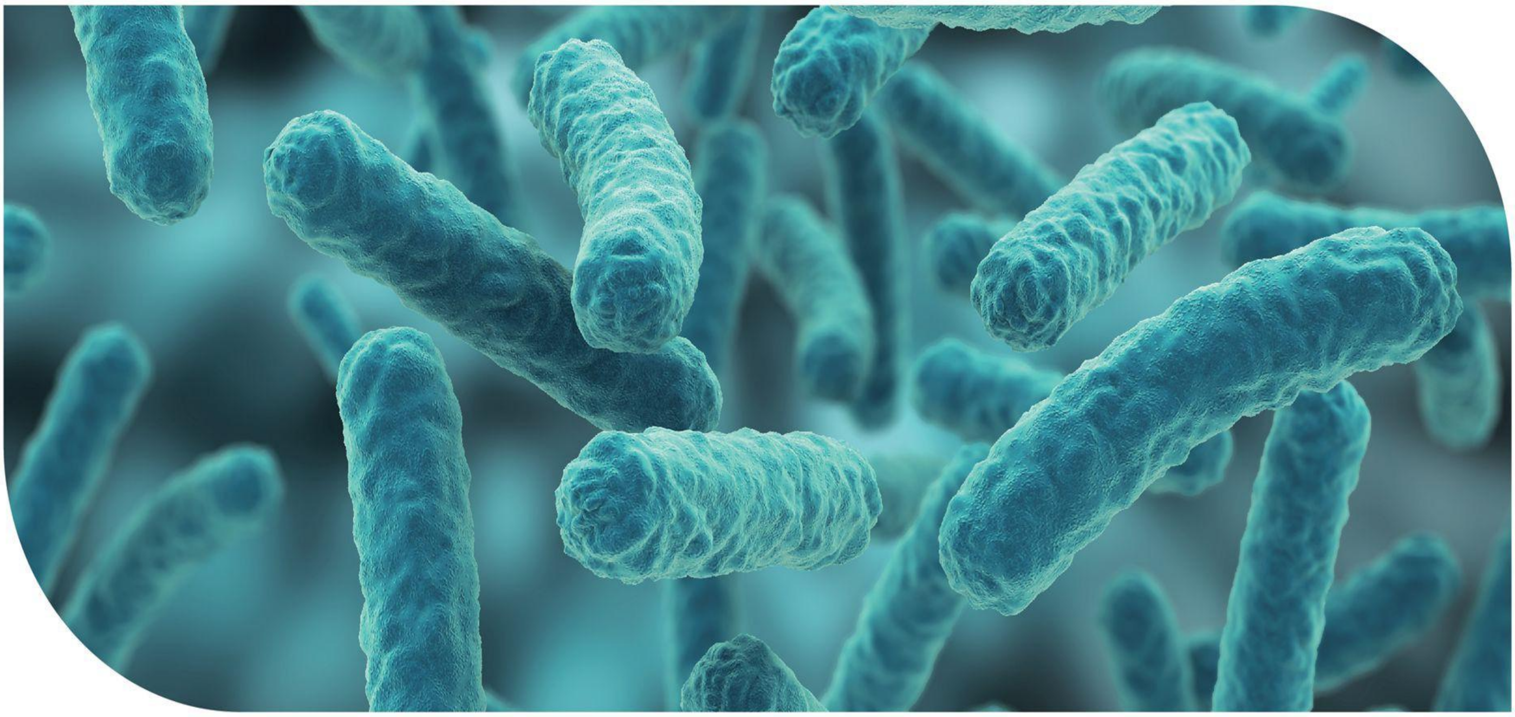

姓名：　　　　　日期：2021-08-24

北京博奥医学检验所

一、样品信息

（一）受检者

姓名：	性别：女
年龄：32岁	住院号/门诊号：-

（二）样品信息

送 检 号：201590002037	样品编号：K2120201_BALF
样品类型：肺泡灌洗液	样品体积：13.5ml
样本取样部位：-	样本取样深度：-
接收日期：2021-08-22	报告日期：2021-08-24 09:18:08

（三）送检信息

送检单位：福建省立医院	送检科室：重症医学三科
送检医生：张颖蕊	

（四）临床信息

临床症状： 重症肺炎，ARDS，肾移植术后 既往病史：CRD-5，肾移植术后
前期检测： 重点关注：病毒,细**菌**,**真菌**,**支**原体/**衣**原体,**耐药基因**
前期**用药**：**美罗**培**南**，**大扶康**

检验人： **审核人：** 报告日期：2021-08-24

二、检测结果

1-检出<高置信度>阳性指标
惠普尔养障体、鲍曼不动杆菌、纹带棒状杆菌、铜绿假单胞菌、产酸克雷伯氏菌、阴道加德纳氏菌、阴道阿托波氏菌、大芬戈尔德菌、葡萄牙棒孢酵母
2-检出<中置信度>阳性指标
近平滑念珠菌、热带假丝酵母
3-检出<低置信度>阳性指标
黏液玫瑰单胞菌

【阳性指标说明】:

1. 惠普尔养障体：检出的惠普尔养障体为革兰氏阳性杆菌，既有可能是呼吸道定植菌，同时也可能为引起惠普尔氏病、心内膜炎、肺炎等的条件致病菌，其具体临床意义需临床医生根据患者临床症状进一步判断。

2. 鲍曼不动杆菌：检出的鲍曼不动杆菌为非发酵革兰阴性、需氧杆菌，广泛存在于自然界，属于条件致病菌，该菌是医院感染的重要病原菌，主要引起呼吸道感染，也可引发菌血症、泌尿系感染、继发性脑膜炎、手术部位感染、呼吸机相关性肺炎等。同时检出耐药基因adeJ、adeB，提示该菌可能为耐药菌，详细耐药信息见本报告耐药基因筛查结果。

3. 纹带棒状杆菌：检出的纹带棒状杆菌是一种革兰氏阳性无芽胞需氧杆菌，主要存在于外界环境以及正常人的皮肤表面，该菌为条件致病菌，可在免疫力低下人群中引起肺炎、菌血症、心内膜炎等。同时检出耐药基因ErmX，提示该菌可能为耐药菌，详细耐药信息见本报告耐药基因筛查结果。

4. 铜绿假单胞菌：检出的铜绿假单胞菌是一种非发酵革兰氏阴性杆菌，该菌广泛分布在环境中，为条件致病菌，当人体抵抗力下降时容易引起感染，可引起烧伤创面感染、肺部感染、泌尿道感染、中耳炎、脑膜炎、败血症等。同时检出耐药基因MexB、mexW，提示该菌可能为耐药菌，详细耐药信息见本报告耐药基因筛查结果。

5. 产酸克雷伯氏菌：检出的产酸克雷伯氏菌是一种革兰氏阴性杆菌，为条件致病菌，可引起肺炎、脑膜炎、菌血症、尿道感染，手术伤口感染等。

6. 阴道加德纳氏菌：检出阴道加德纳氏菌是一种兼性厌氧菌，是革兰氏染色不稳定的细菌菌，为细菌性阴道病的病原菌，该菌可以引起宫颈炎、术后感染、尿道感染等，2014年有研究表明在93名患有尿道炎的男性患者中有15%的患者口腔中检出该菌。该菌在该样本检出的临床意义需临床医生根据患者临床症状进一步判断。

7. 阴道阿托波氏菌：检出的阴道阿托波氏菌是一种革兰氏阳性菌，该菌为阴道菌群成员，可引起阴道感染、菌血症流产、心内膜炎等。

8. 大芬戈尔德菌：检出的大芬戈尔德菌为革兰阳性专性厌氧球菌，是胃肠道和泌尿生殖道的正常菌群，也可以从皮肤和口腔中分离得到，为条件致病菌，有研究表明该菌可引起肺部、软组织脓肿、感染性心内膜炎等。

9. 黏液玫瑰单胞菌：检出的黏液玫瑰单胞菌是一种革兰氏阴性、需氧的小球杆菌，属于玫瑰单胞菌属细菌，常存在于水、土壤等自然环境中，该菌为条件致病菌，可引起菌血症、眼内炎、皮炎等，其具体临床意义需临床医生根据患者临床症状进一步判断。其检出的基因组覆盖度仅为0.06%，为该技术的检测灰区，置信等级为疑似，仅供临床参考。

10. 葡萄牙棒孢酵母：检出的葡萄牙棒孢酵母是一种罕见的机会性病原体，该菌可引起败血症、肾盂肾炎、肺部感染等。

11. 近平滑念珠菌：检出的近平滑念珠菌是一种圆形或者卵圆形的真菌，该菌广泛存在于自然界，为条件致病性真菌，可引起泌尿生殖道感染、肺的深部感染、败血症等。

12. 热带假丝酵母：检出的热带假丝酵母为条件致病菌，可引起皮肤黏膜感染、内脏感染、中枢神经系统感染等。

置信度：根据序列比对，综合评价在样品中鉴定该病原体的可信度。

三、检测结果列表

1. 细菌筛查结果

类型	属			种				
	中文名	拉丁文名	序列数	中文名	拉丁文名	序列数	基因组覆盖度	估测浓度[copies/mL]
G+	-	*Trophery ma*	6100499	惠普尔养障体	*Trophery ma w hipplei*	6100499	925130 bp / 99.77%	1.4E+06
G-	-	*Acinetobacter*	314431	鲍曼不动杆菌	*Acinetobacter bau mannii*	308705	3004073 bp / 72.28%	1.5E+04
G+	-	*Cory nebacteriu m*	161153	纹带棒状杆菌	*Cory nebacteriu m striatu m*	147191	2200174 bp / 77.77%	1.1E+04
G-	-	*Pseu domonas*	96855	铜绿假单胞菌	*Pseu domonas aeru ginosa*	96643	4316384 bp / 62.15%	2.9E+03
G-	-	*Klebsiella*	16590	产酸克雷伯氏菌	*Klebsiella oxy toca*	15974	687093 bp / 11.85%	5.7E+02
G+	-	*Gardnerella*	3324	阴道加德纳氏菌	*Gardnerella v aginalis*	3324	270149 bp / 15.65%	4.0E+02
G+	-	*Atopobiu m*	2889	阴道阿托波氏菌	*Atopobiu m v aginae*	2702	187573 bp / 13.11%	3.9E+02
G-	-	*Roseomonas*	1453	黏液玫瑰单胞菌	*Roseomonas mu cosa*	25	3231 bp / 0.06%	1.0E+00
G+	-	*Finegoldia*	566	大芬戈尔德菌	*Finegoldia magna*	566	40491 bp / 1.99%	5.8E+01

2. 病毒筛查结果

未检出

3. 真菌、寄生虫筛查结果

属			种				
中文名	拉丁文名	序列数	中文名	拉丁文名	序列数	基因组覆盖度	估测浓度[copies/mL]
-	*Clav ispora*	4381	葡萄牙棒孢酵母	*Clav ispora lu sitania e*	4371	393289 bp / 3.25%	7.5E+01
-	*Candida*	189	近平滑念珠菌	*Candida parapsilosi s*	98	11506 bp / 0.09%	1.6E+00
			热带假丝酵母	*Candida tropicalis*	81	9676 bp / 0.07%	1.2E+00

4. 结核分枝杆菌复合群筛查结果

未检出

5. 非结核分枝杆菌筛查结果

未检出

6. 支原体/衣原体/立克次氏体筛查结果

未检出

7. 耐药基因筛查结果		
检测到的耐药基因	基因耐药参考	检出序列数
adeJ [Acinetobacter bau mannii]	大环内酯类;氟喹诺酮类;林可胺类;碳青霉烯;头孢菌素;四环素类;利福霉素;二氨基嘧啶类;利胆醇;青霉烯	312
ErmX [Cory nebacteriu m striatu m]	大环内酯类;林可胺类;链阳霉素	211
adeB [Acinetobacter bau mannii]	甘氨酰环素;四环素类	165
Mex B [Pseu domonas aeru ginosa]	大环内酯类;氟喹诺酮类;单环菌素;碳青霉烯;头孢菌素;头霉素;青霉烷;四环素类;肽类;氨基香豆素;二氨基嘧啶类;磺胺类;利胆醇;青霉烯	80
mex W [Pseu domonas aeru ginosa PAO1]	大环内酯类;氟喹诺酮类;氨基糖苷类;头孢菌素;青霉烷;四环素类;氨基香豆素;二氨基嘧啶类;利胆醇	60

四、测序质控

测序平台	总reads数	用于鉴定的reads平均长度	IC是否检出
BioelectronSeq4000	30000000	151	是

五、检测方法学介绍

（一）检测内容

基于二代测序的宏基因组测序技术，直接对样品中的核酸进行检测，获得样品中微生物的序列信息，无需提前预判感染微生物，无偏向性的鉴定可疑致病微生物。博奥MAPMI TM检测基于BioelectronSeq 4000基因测序仪，可检测病毒（涵盖DNA病毒和RNA病毒）、细菌、真菌、寄生虫共31400个指标以及2500个耐药基因。

（二）检测局限性

1. 本方法与其它检测方法一样，有自身的检测能力和检测范围，本次检测未报告微生物不代表样本中一定不存在致病微生物，并不能排除受检者感染某种病原微生物的可能性，其原因包括但不限于： 1). 样品中病原微生物浓度低于检测限； 2). 病原微生物未被涵盖在检测范围内。
2. 临床研究表明，耐药基因与实际耐药表型并不完全一致，报告中耐药基因检测结果仅供临床参考。

（三）检测结果说明

1. 以上检测结果仅供临床参考，不作为临床诊断唯一依据，如有疑义请在收到结果后七个工作日内与我们联系；
2. 本报告结果仅对本次送检样品负责，报告相关解释需咨询临床医生；
3. 本检测对该结果保密并依法保护受检者隐私，但因受检者个人原因出现信息外泄，本实验室不承担相应责任。

（四）检测流程

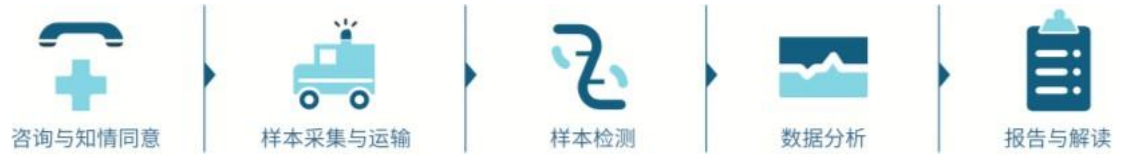

（五）检测范围

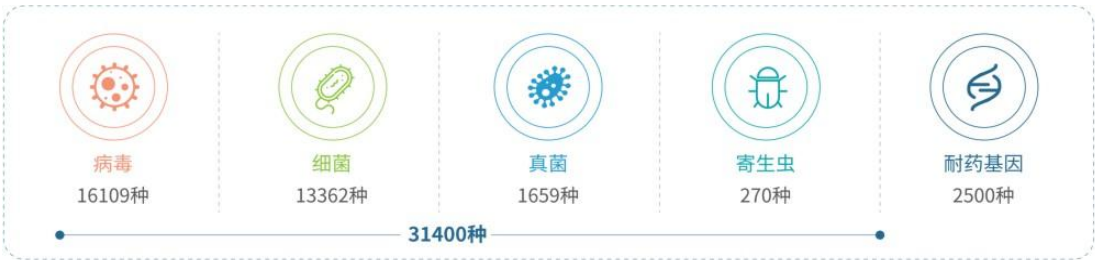

六、参考文献

[1] Wilson MR, Naccache SN, Samayoa E, et al. Actionable diagnosis of neuroleptospirosis by next-generation sequencing. N Engl J Med. 2014;370(25):2408-2417.
[2] Parize P, Muth E, Richaud C, et al. Untargeted next-generation sequencing-based first-line diagnosis of infection in immunocompromised adults: a multicentre, blinded, prospective study. Clin Microbiol Infect. 2017;23(8):574.e1-574.e6.
[3] Quince C, Walker AW, Simpson JT, Loman NJ, Segata N. Shotgun metagenomics, from sampling to analysis [published correction appears in Nat Biotechnol. 2017 Dec 8;35(12):1211]. Nat Biotechnol. 2017;35(9):833-844.
[4] Brown JR, Bharucha T, Breuer J. Encephalitis diagnosis using metagenomics: application of next generation sequencing for undiagnosed cases. J Infect. 2018;76(3):225-240.
[5] Horiba K, Kawada JI, Okuno Y, et al. Comprehensive detection of pathogens in immunocompromised children with bloodstream infections by next-generation sequencing. Sci Rep. 2018;8(1):3784. Published 2018 Feb 28.
[6] Xie Y, Du J, Jin W, et al. Next generation sequencing for diagnosis of severe pneumonia: China, 2010-2018. J Infect. 2019;78(2):158-169.
[7] Wilson MR, Sample HA, Zorn KC, et al. Clinical Metagenomic Sequencing for Diagnosis of Meningitis and Encephalitis. N Engl J Med. 2019;380(24):2327-2340.
[8] Manso CF, Bibby DF, Mohamed H, Brown DWG, Zuckerman M, Mbisa JL. Enhanced Detection of DNA Viruses in the Cerebrospinal Fluid of Encephalitis Patients Using Metagenomic Next-Generation Sequencing. Front Microbiol. 2020;11:1879. Published 2020 Aug 12.
[9] Selway CA, Eisenhofer R, Weyrich LS. Microbiome applications for pathology: challenges of low microbial biomass samples during diagnostic testing. J Pathol Clin Res. 2020 Apr;6(2):97-106.
[10] Li N, Cai Q, Miao Q, et al. High-Throughput Metagenomics for Identification of Pathogens in the Clinical Settings. Small Methods. 2021 Jan 4;5(1):2000792.

附录一-[阳性指标详细信息]

（一）阳性细菌详细技术信息

排名：1	种名: *Trophery ma w hipplei*	种分类号：2039
检出reads数：6100499 覆盖度：99.77%		

\# 技术性注释 #
\# 共有 6100499 条reads命中“2039（ *Trophery ma w hipplei* ）”
\# 命中的物种基因组总长度为 927303 bp，测到的该物种序列拼接后总长度为 925130 bp，覆盖度为 99.77%
\# 测到的该物种序列的总碱基数为 938381509 bp，测到的该物种序列拼接后总长度为 925130 bp，平均深度为 1014.3X

命中参考序列：Tropheryma_whipplei_str._Twist

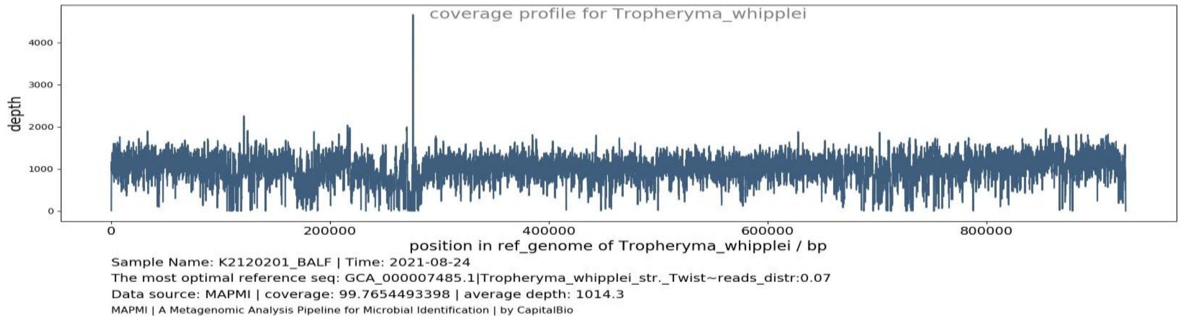

排名：2	种名: *Acinetobacter bau mannii*	种分类号：470
检出reads数：308705 覆盖度：72.28%		

\# 技术性注释 #
\# 共有 308705 条reads命中“470（ *Acinetobacter bau mannii* ”
\# 命中的物种基因组总长度为 4156196 bp，测到的该物种序列拼接后总长度为 3004073 bp，覆盖度为 72.28%
\# 测到的该物种序列的总碱基数为 29901343 bp，测到的该物种序列拼接后总长度为 3004073 bp，平均深度为 10.0X

命中参考序列：Acinetobacter_baumannii_UH10707

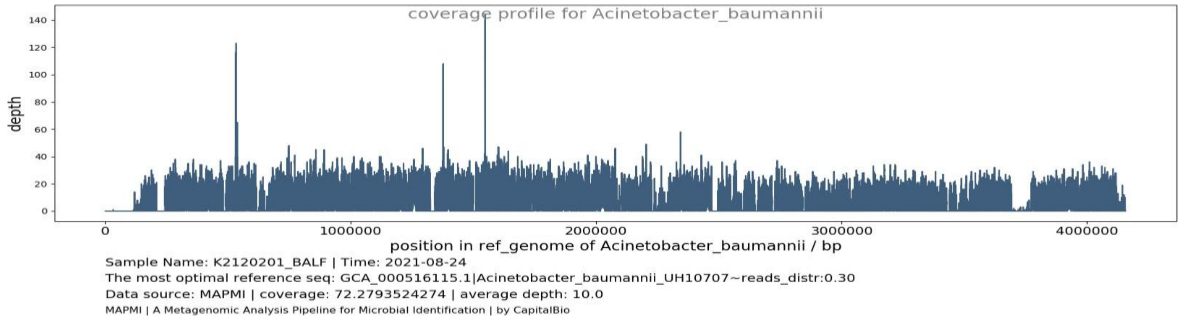

排名：3	种名 *Cory nebacteriu m striatu m*	种分类号：43770

检出reads数：147191 覆盖度：77.77%

\# 技术性注释 #
\# 共有 147191 条reads命中“43770（*Cory nebacteriu m striatu m*”
\# 命中的物种基因组总长度为 2828991 bp，测到的该物种序列拼接后总长度为 2200174 bp，覆盖度为 77.77%
\# 测到的该物种序列的总碱基数为 21600562 bp，测到的该物种序列拼接后总长度为 2200174 bp，平均深度为 9.8X

命中参考序列：Corynebacterium_striatum_ATCC_6940

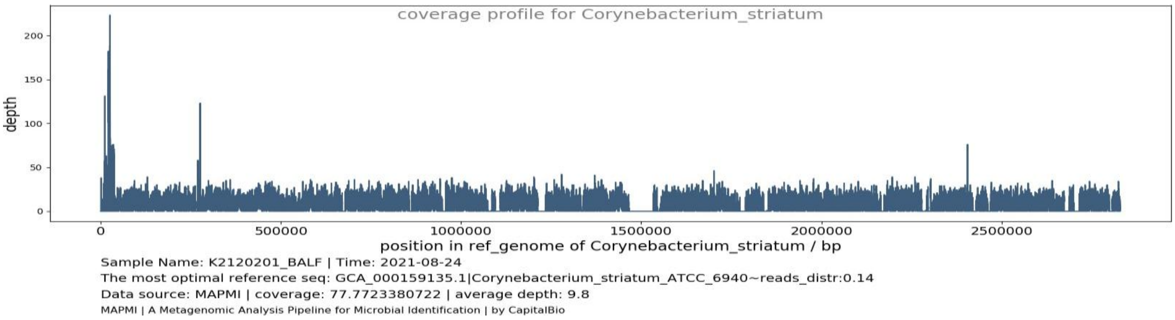

排名：4	种名: *Pseu domonas aeru ginosa*	种分类号：287

检出reads数：96643 覆盖度：62.15%

\# 技术性注释 #
\# 共有 96643 条reads命中“287（*Pseu domonas aeru ginosa* ”
\# 命中的物种基因组总长度为 6944930 bp，测到的该物种序列拼接后总长度为 4316384 bp，覆盖度为 62.15%
\# 测到的该物种序列的总碱基数为 12942099 bp，测到的该物种序列拼接后总长度为 4316384 bp，平均深度为 3.0X

命中参考序列：Pseudomonas_aeruginosa_PA96

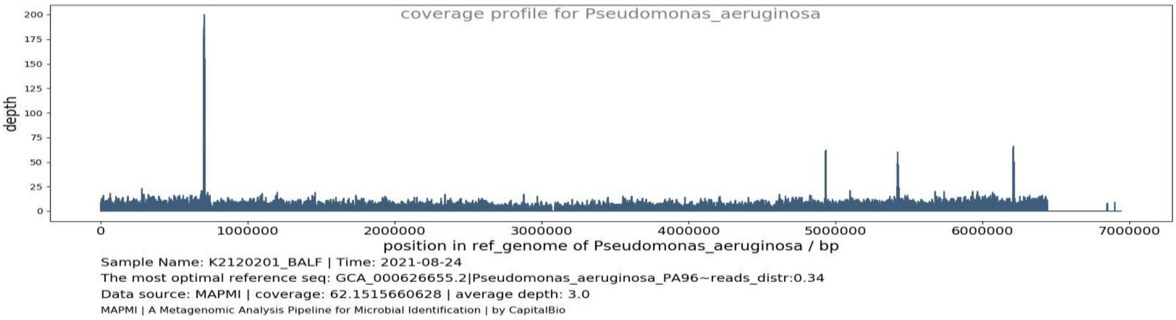

排名：5	种: *Klebsiella oxy toca*	种分类号：571

检出reads数：15974 覆盖度：11.85%

技术性注释
共有 15974 条reads命中“571 (*Klebsiella oxy toca* ”
命中的物种基因组总长度为 5799592 bp，测到的该物种序列拼接后总长度为 687093 bp，覆盖度为 11.85%
测到的该物种序列的总碱基数为 1997578 bp，测到的该物种序列拼接后总长度为 687093 bp，平均深度为 2.9X

命中参考序列：Klebsiella_oxytoca_G54

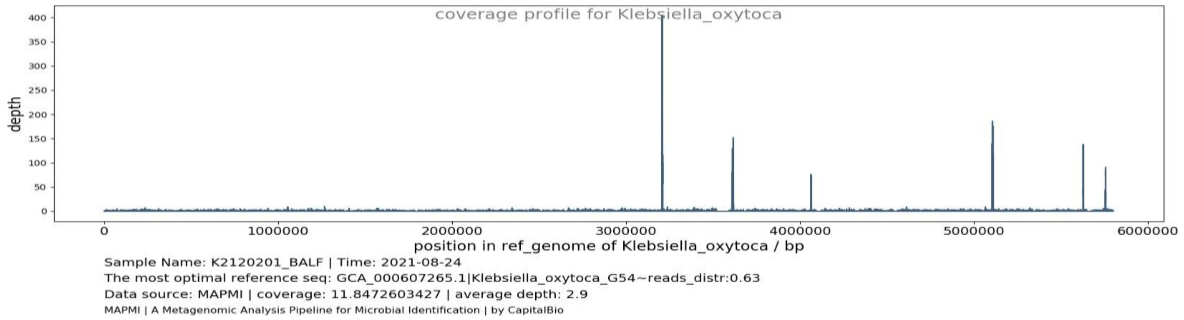

排名：6	种名: *Gardnerella v aginalis*	种分类号：2702

检出reads数：3324 覆盖度：15.65%

技术性注释
共有 3324 条reads命中“2702 (*Gardnerella v aginalis*)”
命中的物种基因组总长度为 1726519 bp，测到的该物种序列拼接后总长度为 270149 bp，覆盖度为 15.65%
测到的该物种序列的总碱基数为 423462 bp，测到的该物种序列拼接后总长度为 270149 bp，平均深度为 1.6X

命中参考序列：Gardnerella_vaginalis_HMP9231

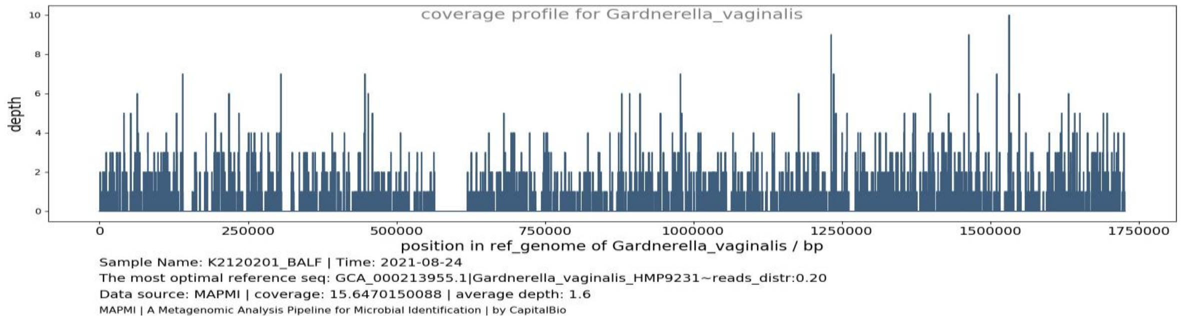

排名：7	种名: *Atopobiu m v aginae*	种分类号：82135
检出reads数：2702 ｜ 覆盖度：13.11%		

技术性注释
共有 2702 条reads命中“82135 (*Atopobiu m v aginae*)”
命中的物种基因组总长度为 1430526 bp，测到的该物种序列拼接后总长度为 187573 bp，覆盖度为 13.11%
测到的该物种序列的总碱基数为 306017 bp，测到的该物种序列拼接后总长度为 187573 bp，平均深度为 1.6X

命中参考序列：Atopobium_vaginae_DSM_15829

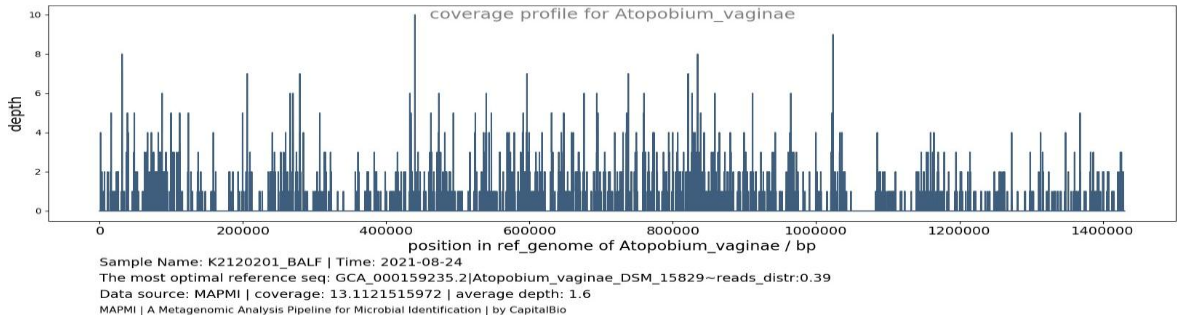

排名：8	种名: *Roseomonas mu cosa*	种分类号：207340
检出reads数：25 ｜ 覆盖度：0.06%		

技术性注释
共有 25 条reads命中“207340 (*Roseomonas mu cosa*)”
命中的物种基因组总长度为 4996294 bp，测到的该物种序列拼接后总长度为 3231 bp，覆盖度为 0.06% # 测到的该物种序列的总碱基数为 3464 bp，测到的该物种序列拼接后总长度为 3231 bp，平均深度为 1.1X

命中参考序列：Roseomonas_mucosa

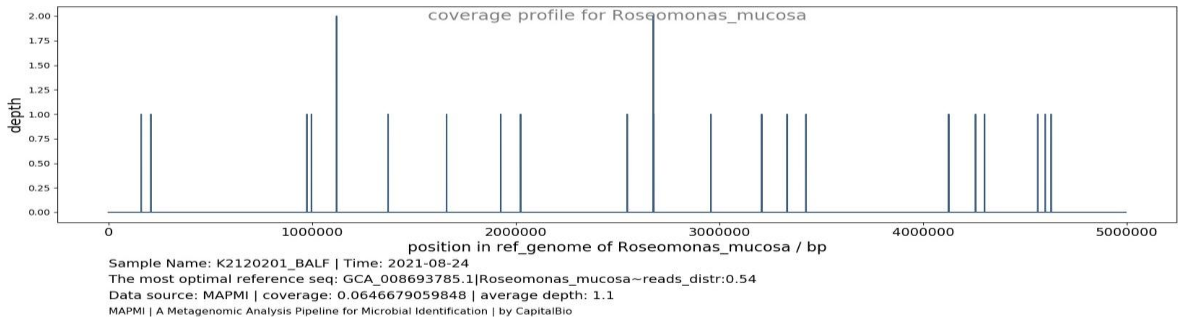

排名：9	种名: *Finegoldia magna*	种分类号：1260
检出reads数：566 ｜ 覆盖度：1.99%		

技术性注释
共有 566 条reads命中“1260 (*Finegoldia magna* ”
命中的物种基因组总长度为 2032717 bp，测到的该物种序列拼接后总长度为 40491 bp，覆盖度为 1.99%
测到的该物种序列的总碱基数为 53797 bp，测到的该物种序列拼接后总长度为 40491 bp，平均深度为 1.3X

命中参考序列：Finegoldia_magna_SY403409CC001050417

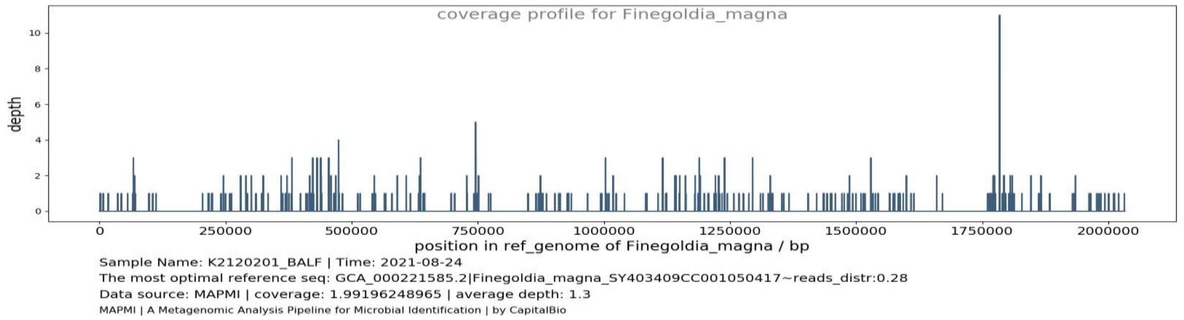

（二）阳性病毒详细技术信息

未检测到相关病毒。

（三）阳性真菌、寄生虫详细技术信息

排名：1	种名: *Clav ispora lu sitaniae*	种分类号：36911
检出reads数：4371 ｜ 覆盖度：3.25%		

技术性注释
共有 4371 条reads命中“36911 (*Clav ispora lu sitaniae*)”
命中的物种基因组总长度为 12115052 bp，测到的该物种序列拼接后总长度为 393289 bp，覆盖度为 3.25%
测到的该物种序列的总碱基数为 596214 bp，测到的该物种序列拼接后总长度为 393289 bp，平均深度为 1.5X

命中参考序列：Clavispora_lusitaniae_ATCC_42720

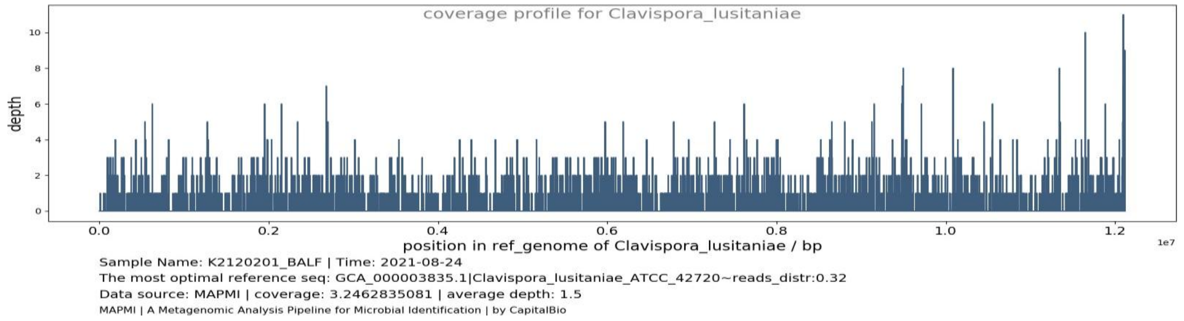

排名：2	种名: *Candida parapsilosis*	种分类号：5480
检出reads数：98 ｜ 覆盖度：0.09%		

技术性注释
共有 98 条reads命中“5480 (*Candida parapsilosis* ”
命中的物种基因组总长度为 13030334 bp，测到的该物种序列拼接后总长度为 11506 bp，覆盖度为 0.09% # 测到的该物种序列的总碱基数为 14639 bp，测到的该物种序列拼接后总长度为 11506 bp，平均深度为 1.3X

命中参考序列：Candida_parapsilosis

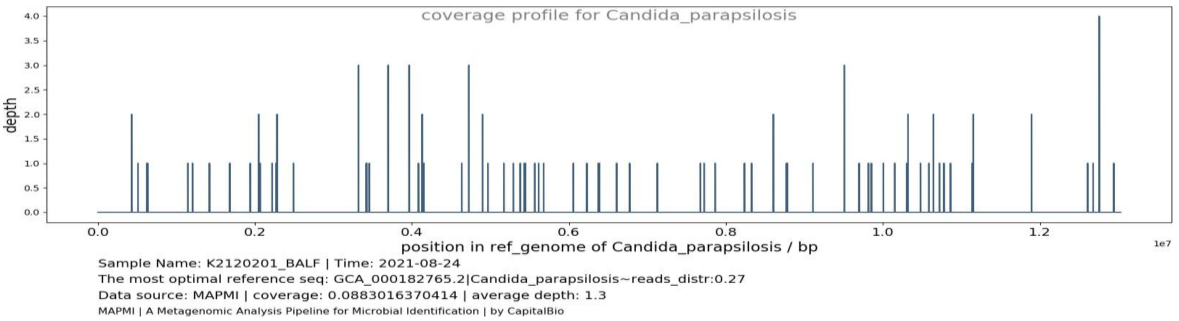

排名：3	种名: *Candida tropicalis*	种分类号：5482
检出reads数：81 ｜ 覆盖度：0.07%		

技术性注释
共有 81 条reads命中“5482 (*Candida tropicalis* ”
命中的物种基因组总长度为 14630599 bp，测到的该物种序列拼接后总长度为 9676 bp，覆盖度为 0.07% # 测到的该物种序列的总碱基数为 12254 bp，测到的该物种序列拼接后总长度为 9676 bp，平均深度为 1.3X

命中参考序列：Candida_tropicalis_MYA-3404

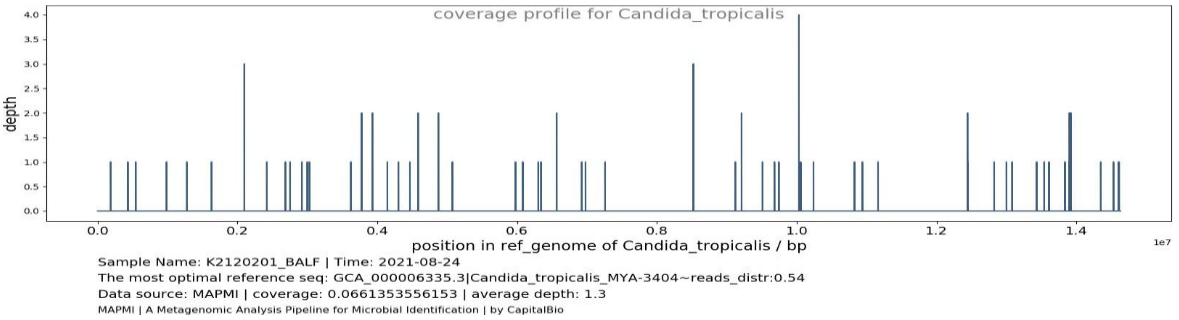

（四）阳性结核分枝杆菌复合群详细技术信息

未检测到相关结核分枝杆菌复合群。

（五）阳性非结核分枝杆菌详细技术信息

未检测到相关非结核分枝杆菌。

（六）阳性支原体/衣原体/立克次氏体详细技术信息

未检测到相关支原体/衣原体/立克次氏体。

（七）阳性耐药基因详细技术信息

排名：1	种名: *adeJ [Acinetobacter bau mannii]*	种分类号：AR4985794

检出reads数：312 ｜ 覆盖度：99.78%

技术性注释
共有 312 条reads命中“AR4985794（ *adeJ [Acinetobacter bau mannii]* ”
命中的物种基因组总长度为 3179 bp，测到的该物种序列拼接后总长度为 3174 bp，覆盖度为 99.78%
测到的该物种序列的总碱基数为 48203 bp，测到的该物种序列拼接后总长度为 3174 bp，平均深度为 15.2X

命中参考序列：adeJ_[Acinetobacter_baumannii]

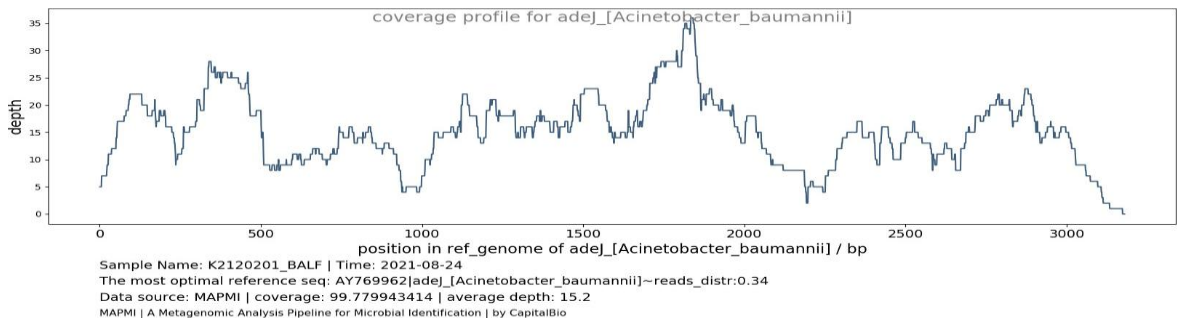

排名：2	种名: *ErmX [Cory nebacteriu m striatu m]*	种分类号：AR4985609

检出reads数：211 ｜ 覆盖度：99.09%

技术性注释
共有 211 条reads命中“AR4985609（ *ErmX [Cory nebacteriu m striatu m]* ”
命中的物种基因组总长度为 764 bp，测到的该物种序列拼接后总长度为 759 bp，覆盖度为 99.09%
测到的该物种序列的总碱基数为 33382 bp，测到的该物种序列拼接后总长度为 759 bp，平均深度为 44.0X

命中参考序列：ErmX_[Corynebacterium_striatum]

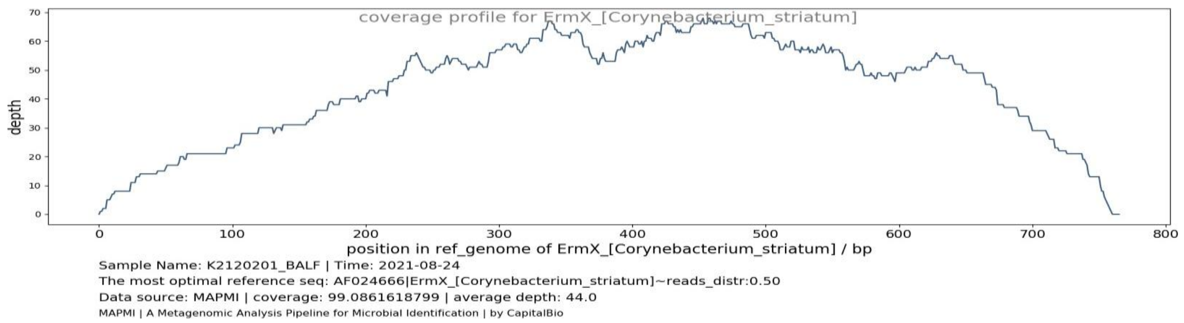

排名：3	种名: *adeB [Acinetobacter bau mannii]*	种分类号：AR4985788

检出reads数：165　覆盖度：96.11%

技术性注释
共有 165 条reads命中“AR4985788（*adeB [Acinetobacter bau mannii]*”
命中的物种基因组总长度为 3109 bp，测到的该物种序列拼接后总长度为 2990 bp，覆盖度为 96.11%
测到的该物种序列的总碱基数为 25423 bp，测到的该物种序列拼接后总长度为 2990 bp，平均深度为 8.5X

命中参考序列：adeB_[Acinetobacter_baumannii]

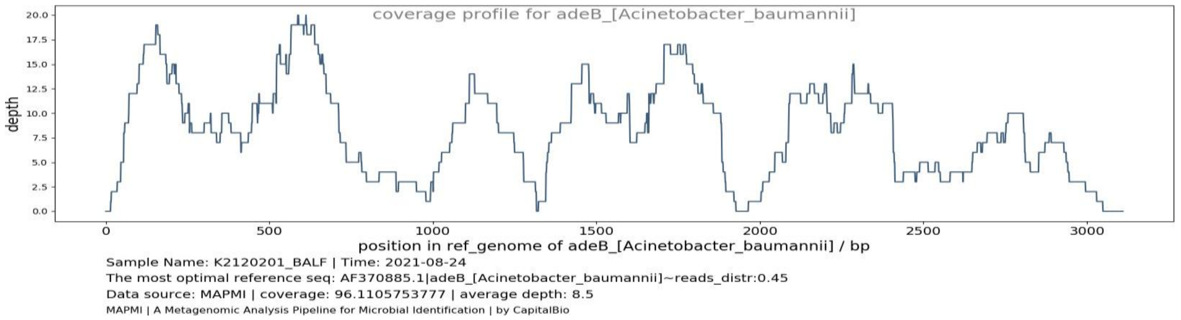

排名：4	种名: *Mex B [Pseu domonas aeru ginosa]*	种分类号：AR4985391

检出reads数：80　覆盖度：91.92%

技术性注释
共有 80 条reads命中“AR4985391（*Mex B [Pseu domonas aeru ginosa]*”
命中的物种基因组总长度为 3143 bp，测到的该物种序列拼接后总长度为 2891 bp，覆盖度为 91.92%
测到的该物种序列的总碱基数为 11429 bp，测到的该物种序列拼接后总长度为 2891 bp，平均深度为 4.0X

命中参考序列：MexB_[Pseudomonas_aeruginosa]

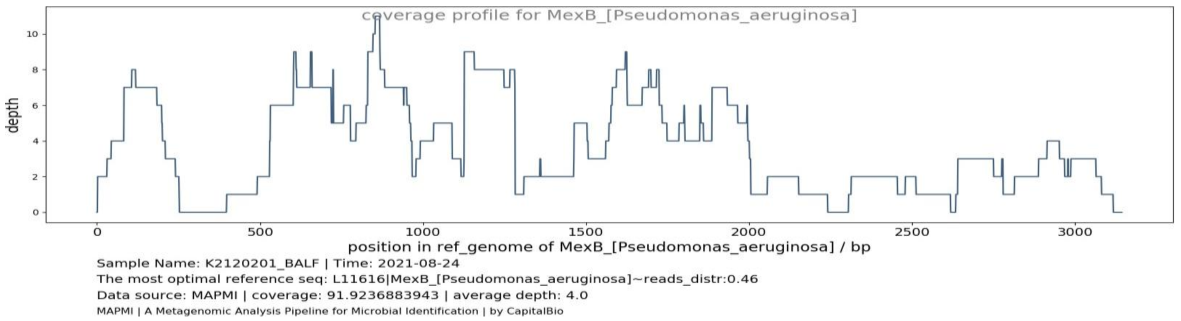

排名：5	种名: *mex W [Pseu domonas aeru ginosa PAO1]*	种分类号：AR4988044
检出reads数：60 ｜ 覆盖度：91.14%		

技术性注释

共有 60 条reads命中“AR4988044（*mex W [Pseu domonas aeru ginosa PAO1]*”

命中的物种基因组总长度为 3058 bp，测到的该物种序列拼接后总长度为 2789 bp，覆盖度为 91.14%

测到的该物种序列的总碱基数为 9185 bp，测到的该物种序列拼接后总长度为 2789 bp，平均深度为 3.3X

命中参考序列：mexW_[Pseudomonas_aeruginosa_PAO1]

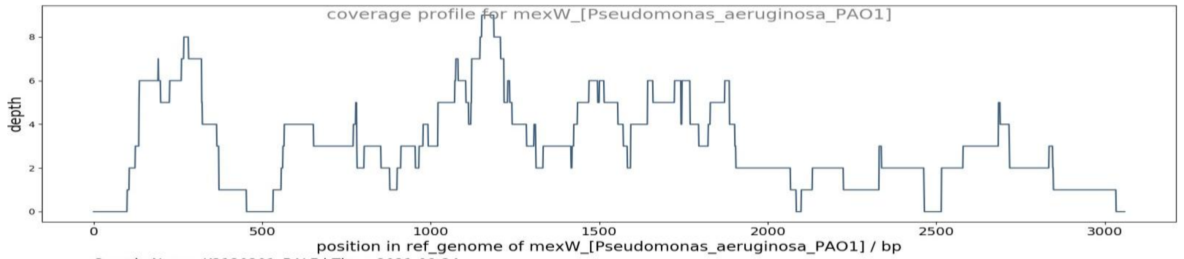

Sample Name: K2120201_BALF | Time: 2021-08-24
The most optimal reference seq: NC_002516.2_7|mexW_[Pseudomonas_aeruginosa_PAO1]~reads_distr:0.56
Data source: MAPMI | coverage: 91.1437908497 | average depth: 3.3
MAPMI | A Metagenomic Analysis Pipeline for Microbial Identification | by CapitalBio

附录二-[疑似病原检测结果列表]

类型	属	种
细菌	未检出	
病毒	未检出	
真菌	未检出	
寄生虫	未检出	

附录三-[疑似定植菌检测结果列表]

类型	属			种			来源
	中文名	拉丁文名	序列数	中文名	拉丁文名	序列数	
G+	-	*Cory nebacteriu m*	161153	棒状杆菌属细菌	*Cory nebacteriu m simu lans*	3080	呼吸道
				拥挤棒状杆菌	*Cory nebacteriu m accolens*	83	呼吸道
G+	-	*Atopobiu m*	2889	极小奇异菌	*Atopobiu m parv u lu m*	119	口腔
				奇异菌属细菌	*Atopobiu m rimae*	50	口腔
G+	-	*Streptococcu s*	743	咽峡炎链球菌	*Streptococcu s anginosu s*	38	口腔
G+	-	*Parv imonas*	76	微单胞菌属细菌	*Parv imonas micra*	61	口腔
G+	-	*Dolosigranu lu m*	58	懒惰狡诈球菌	*Dolosigranu lu m pigru m*	55	呼吸道
病毒	未检出						
真菌	未检出						
寄生虫	未检出						
