# Supplementary material for: Tropheryma whipplei pneumonia: a retrospective case series of nine patients with treatment response
Source: Front Med (Lausanne). 2026 Jun 29;13:1883057. doi: 10.3389/fmed.2026.1883057 (PMC13357807; doi:10.3389/fmed.2026.1883057)
Supplement: Supplementary file 2 [file Data_Sheet_2.PDF]

MAPMI™ 超广谱病原微生物 mNGS 检测报告

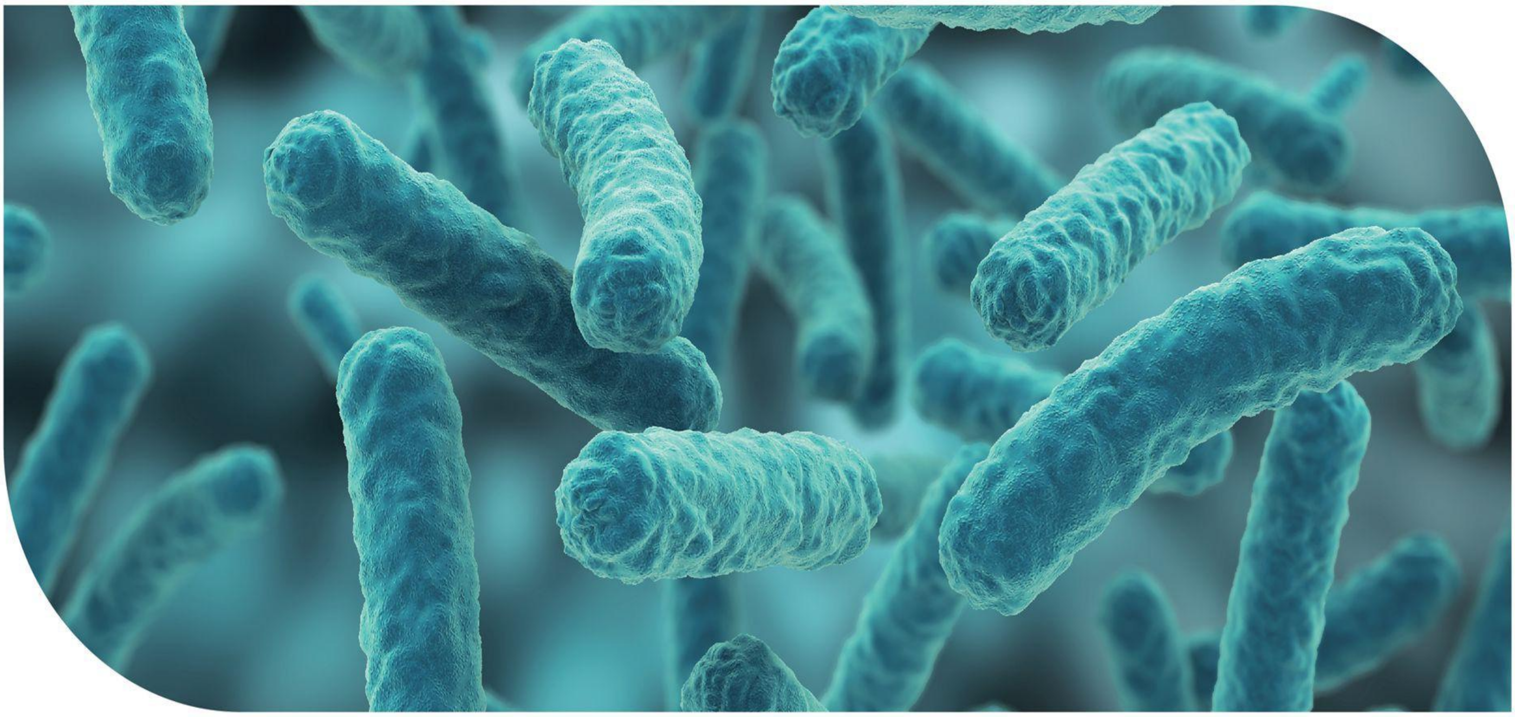

姓名：　　　　　　日期：2021-09-02

北京博奥医学检验所

一、样品信息

（一）受检者

姓名：	性别：女
年龄：32岁	住院号/门诊号：

（二）样品信息

送 检 号：201590002941	样品编号：K2121001_BALF
样品类型：肺泡灌洗液	样品体积：12ml
样本取样部位：-	样本取样深度：-
接收日期：2021-08-31	报告日期：2021-09-02 11:58:32

（三）送检信息

送检单位：福建省立医院	送检科室：重症三科
送检医生：颜婉莉	

（四）临床信息

临床症状：发热，气促 重症肺炎 既往病史：CKD5期，肾移植术后
前期检测：培养：铜绿假单胞 重点**关注**：病**毒**,**细菌**,**真菌**
前期**用药**：**美罗**，**环丙**，**稳可**信，**大扶康**

检验人：

审核人：

报告日期：2021-09-02

二、检测结果

1-检出<高置信度>阳性指标
人疱疹病毒5型（巨细胞病毒）、屎肠球菌、解甘露醇罗尔斯顿菌、纹带棒状杆菌
2-检出<中置信度>阳性指标
铜绿假单胞菌
3-检出<低置信度>阳性指标
人疱疹病毒7型

【阳性指标说明】：

1. 人疱疹病毒5型（巨细胞病毒）：检出的人疱疹病毒5型（巨细胞病毒）为DNA病毒，该病毒在人群中分布广泛，通常为隐性感染，一般免疫力低下者感染后可引起肺炎、脑膜炎和肝炎等。

2. 人疱疹病毒7型：检出的人疱疹病毒7型为DNA病毒，主要通过唾液传播，可能与多种疾病相关，如幼儿急疹、肝炎、神经系统损害和移植后并发症等。其检出的序列仅有4条，为该技术的检测灰区，置信等级为疑似，仅供临床参考。

3. 屎肠球菌：检出的屎肠球菌是一种革兰氏阳性球菌，该菌可寄生在动物和人肠道、呼吸道及女性生殖道，为条件致病菌，可引起败血症、化脓性脑膜炎、下呼吸道感染、尿路感染、腹泻以及伤口感染等。同时检出耐药基因ErmB、efmA、AAC(6')-Ii，提示该菌可能为耐药菌，详细耐药信息见本报告耐药基因筛查结果。

4. 解甘露醇罗尔斯顿菌：检出的解甘露醇罗尔斯顿菌是一种革兰氏阴性菌，是院内感染少见的机会致病菌，可引起腹腔感染、肺炎、菌血症和脑膜炎等。

5. 纹带棒状杆菌：检出的纹带棒状杆菌是一种革兰氏阳性无芽胞需氧杆菌，主要存在于外界环境以及正常人的皮肤表面，该菌为条件致病菌，可在免疫力低下人群中引起肺炎、菌血症、心内膜炎等。

6. 铜绿假单胞菌：检出的铜绿假单胞菌是一种非发酵革兰氏阴性杆菌，该菌广泛分布在环境中，为条件致病菌，当人体抵抗力下降时容易引起感染，可引起烧伤创面感染、肺部感染、泌尿道感染、中耳炎、脑膜炎、败血症等。

置信度：根据序列比对，综合评价在样品中鉴定该病原体的可信度。

三、检测结果列表

1. 细菌筛查结果

类型	属			种				
	中文名	拉丁文名	序列数	中文名	拉丁文名	序列数	基因组覆盖度	估测浓度[copies/mL]
G+	-	*Enterococcu s*	1074688	屎肠球菌	*Enterococcu s faeciu m*	1073445	2741077 bp / 86.79%	1.2E+05
G-	-	*Ralstonia*	542518	解甘露醇罗尔斯顿菌	*Ralstonia mannitolil y tica*	524073	4128438 bp / 81.39%	3.5E+04
G+	-	*Cory nebacteriu m*	15754	纹带棒状杆菌	*Cory nebacteriu m striatu m*	10787	679306 bp / 24.01%	1.3E+03
G-	-	*Pseu domonas*	3367	铜绿假单胞菌	*Pseu domonas aeru ginosa*	1137	15189 bp / 0.22%	5.6E+01

2. 病毒筛查结果

属			种				
中文名	拉丁文名	序列数	中文名	拉丁文名	序列数	基因组覆盖度	估测浓度[copies/mL]
-	*Cy tomegalov iru s*	160	人疱疹病毒5型（巨细胞病毒）	*Hu man betaherpes v iru s 5*	31	3237 bp / 1.37%	1.8E+01
-	*Roseolov iru s*	5	人疱疹病毒7型	*Hu man betaherpes v iru s 7*	4	373 bp / 0.24%	3.5E+00

3. 真菌、寄生虫筛查结果

未检出

4. 结核分枝杆菌复合群筛查结果

未检出

5. 非结核分枝杆菌筛查结果

未检出

6. 支原体/衣原体/立克次氏体筛查结果

未检出

7. 耐药基因筛查结果

检测到的耐药基因	基因耐药参考	检出序列数
ErmB [Enterococcu s faeciu m]	链阳霉素	1241
efmA [Enterococcu s faeciu m]	大环内酯类;氟喹诺酮类	364
AAC(6')-Ii [Enterococcu s faeciu m]	氨基糖苷类	110

四、测序质控

测序平台	总reads数	用于鉴定的reads平均长度	IC是否检出
BioelectronSeq4000	18418781	131	是

五、检测方法学介绍

（一）检测内容

基于二代测序的宏基因组测序技术，直接对样品中的核酸进行检测，获得样品中微生物的序列信息，无需提前预判感染微生物，无偏向性的鉴定可疑致病微生物。博奥MAPMI^TM检测基于BioelectronSeq 4000基因测序仪，可检测病毒（涵盖DNA病毒和RNA病毒）、细菌、真菌、寄生虫共31400个指标以及2500个耐药基因。

（二）检测局限性

1. 本方法与其它检测方法一样，有自身的检测能力和检测范围，本次检测未报告微生物不代表样本中一定不存在致病微生物，并不能排除受检者感染某种病原微生物的可能性，其原因包括但不限于：1). 样品中病原微生物浓度低于检测限；2). 病原微生物未被涵盖在检测范围内。
2. 临床研究表明，耐药基因与实际耐药表型并不完全一致，报告中耐药基因检测结果仅供临床参考。

（三）检测结果说明

1. 以上检测结果仅供临床参考，不作为临床诊断唯一依据，如有疑义请在收到结果后七个工作日内与我们联系；
2. 本报告结果仅对本次送检样品负责，报告相关解释需咨询临床医生；
3. 本检测对该结果保密并依法保护受检者隐私，但因受检者个人原因出现信息外泄，本实验室不承担相应责任。

（四）检测流程

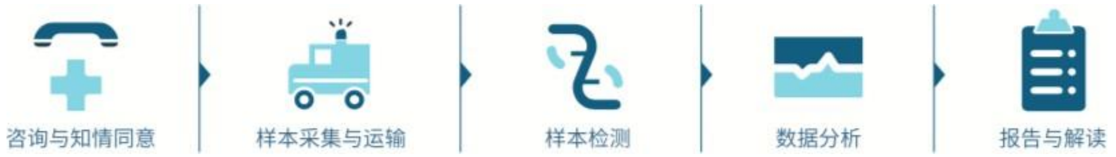

（五）检测范围

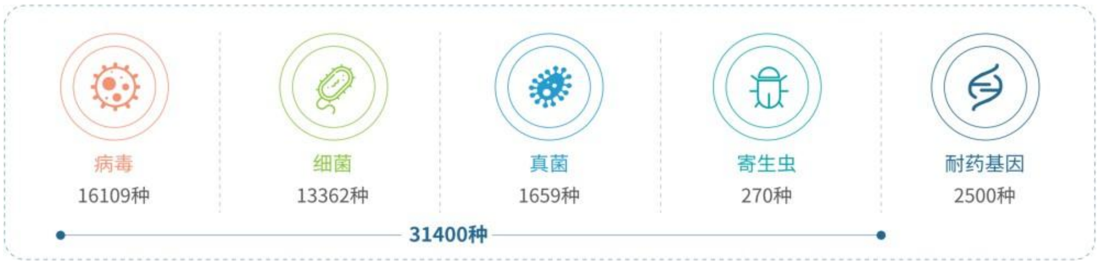

六、参考文献

[1] Wilson MR, Naccache SN, Samayoa E, et al. Actionable diagnosis of neuroleptospirosis by next-generation sequencing. N Engl J Med. 2014;370(25):2408-2417.
[2] Parize P, Muth E, Richaud C, et al. Untargeted next-generation sequencing-based first-line diagnosis of infection in immunocompromised adults: a multicentre, blinded, prospective study. Clin Microbiol Infect. 2017;23(8):574.e1-574.e6.
[3] Quince C, Walker AW, Simpson JT, Loman NJ, Segata N. Shotgun metagenomics, from sampling to analysis [published correction appears in Nat Biotechnol. 2017 Dec 8;35(12):1211]. Nat Biotechnol. 2017;35(9):833-844.
[4] Brown JR, Bharucha T, Breuer J. Encephalitis diagnosis using metagenomics: application of next generation sequencing for undiagnosed cases. J Infect. 2018;76(3):225-240.
[5] Horiba K, Kawada JI, Okuno Y, et al. Comprehensive detection of pathogens in immunocompromised children with bloodstream infections by next-generation sequencing. Sci Rep. 2018;8(1):3784. Published 2018 Feb 28.
[6] Xie Y, Du J, Jin W, et al. Next generation sequencing for diagnosis of severe pneumonia: China, 2010-2018. J Infect. 2019;78(2):158-169.
[7] Wilson MR, Sample HA, Zorn KC, et al. Clinical Metagenomic Sequencing for Diagnosis of Meningitis and Encephalitis. N Engl J Med. 2019;380(24):2327-2340.
[8] Manso CF, Bibby DF, Mohamed H, Brown DWG, Zuckerman M, Mbisa JL. Enhanced Detection of DNA Viruses in the Cerebrospinal Fluid of Encephalitis Patients Using Metagenomic Next-Generation Sequencing. Front Microbiol. 2020;11:1879. Published 2020 Aug 12.
[9] Selway CA, Eisenhofer R, Weyrich LS. Microbiome applications for pathology: challenges of low microbial biomass samples during diagnostic testing. J Pathol Clin Res. 2020 Apr;6(2):97-106.
[10] Li N, Cai Q, Miao Q, et al. High-Throughput Metagenomics for Identification of Pathogens in the Clinical Settings. Small Methods. 2021 Jan 4;5(1):2000792.

附录一-[阳性指标详细信息]

（一）阳性细菌详细技术信息

排名：1	种名: *Enterococcu s faeciu m*	种分类号：1352
检出reads数：1073445 覆盖度：86.79%		

技术性注释
共有 1073445 条reads命中"1352（*Enterococcu s faeciu m* "
命中的物种基因组总长度为 3158418 bp，测到的该物种序列拼接后总长度为 2741077 bp，覆盖度为 86.79%
测到的该物种序列的总碱基数为 123789633 bp，测到的该物种序列拼接后总长度为 2741077 bp，平均深度为 45.2X

命中参考序列：Enterococcus_faecium

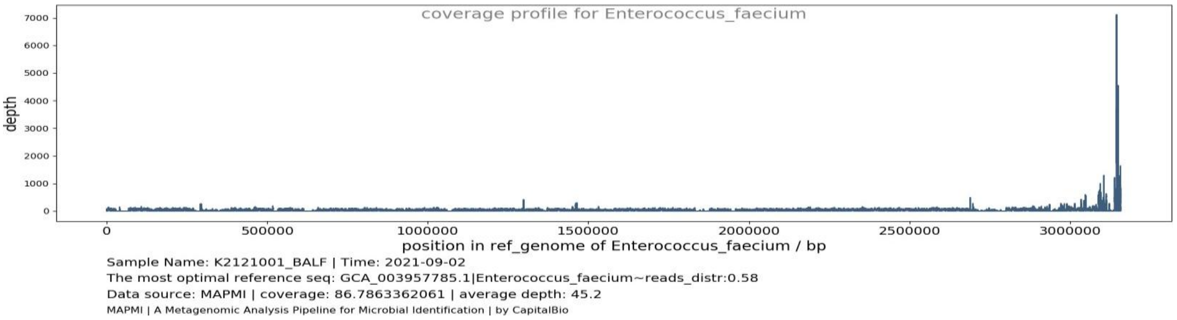

排名：2	种名: *Ralstonia mannitolily tica*	种分类号：105219
检出reads数：524073 覆盖度：81.39%		

技术性注释
共有 524073 条reads命中"105219（*Ralstonia mannitolily tica* "
命中的物种基因组总长度为 5072301 bp，测到的该物种序列拼接后总长度为 4128438 bp，覆盖度为 81.39%
测到的该物种序列的总碱基数为 68521880 bp，测到的该物种序列拼接后总长度为 4128438 bp，平均深度为 16.6X

命中参考序列：Ralstonia_mannitolilytica

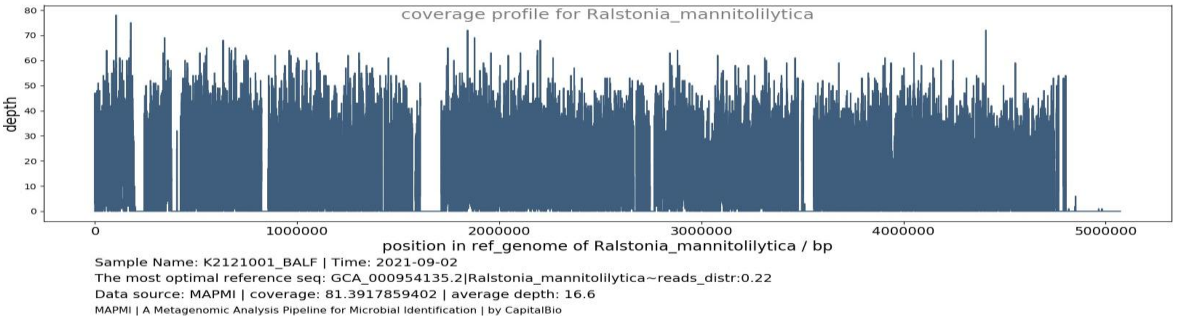

排名：3	种名：*Cory nebacteriu m striatu m*	种分类号：43770
检出reads数：10787 覆盖度：24.01%		
# 技术性注释 #<br># 共有 10787 条reads命中“43770（*Cory nebacteriu m striatu m*）”<br># 命中的物种基因组总长度为 2828991 bp，测到的该物种序列拼接后总长度为 679306 bp，覆盖度为 24.01%<br># 测到的该物种序列的总碱基数为 1379051 bp，测到的该物种序列拼接后总长度为 679306 bp，平均深度为 2.0X		
命中参考序列：Corynebacterium_striatum_ATCC_6940		

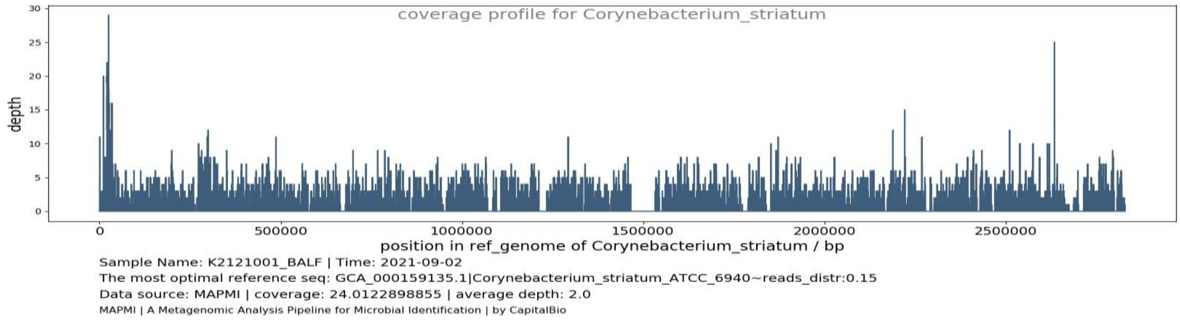

排名：4	种名：*Pseu domonas aeru ginosa*	种分类号：287
检出reads数：1137 覆盖度：0.22%		
# 技术性注释 #<br># 共有 1137 条reads命中“287（*Pseu domonas aeru ginosa*）”<br># 命中的物种基因组总长度为 6944930 bp，测到的该物种序列拼接后总长度为 15189 bp，覆盖度为 0.22%<br># 测到的该物种序列的总碱基数为 23787 bp，测到的该物种序列拼接后总长度为 15189 bp，平均深度为 1.6X		
命中参考序列：Pseudomonas_aeruginosa_PA96		

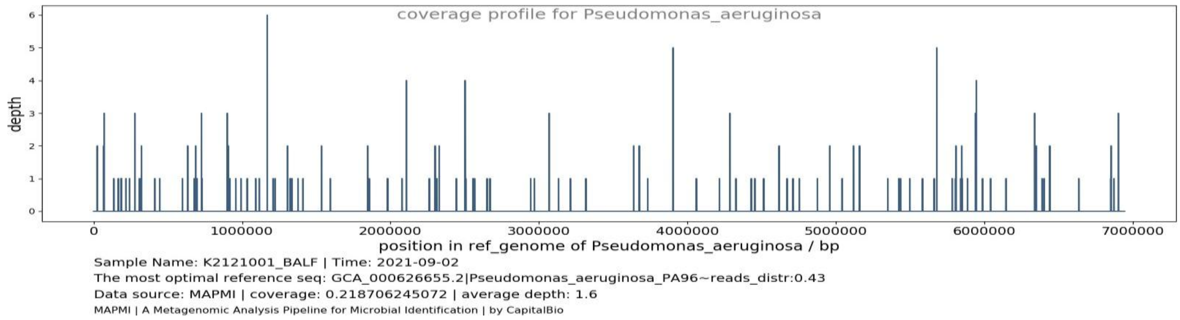

（二）阳性病毒详细技术信息

排名：1	种名：*Hu man betaherpesv iru s 5*	种分类号：10359

检出reads数：31　　覆盖度：1.37%

技术性注释
共有 31 条reads命中"10359 (*Hu man betaherpesv iru s 5*)"
命中的物种基因组总长度为 235775 bp，测到的该物种序列拼接后总长度为 3237 bp，覆盖度为 1.37%
测到的该物种序列的总碱基数为 3982 bp，测到的该物种序列拼接后总长度为 3237 bp，平均深度为 1.2X

命中参考序列：Human_herpesvirus_5_strain_BE/38/2011,_complete_genome

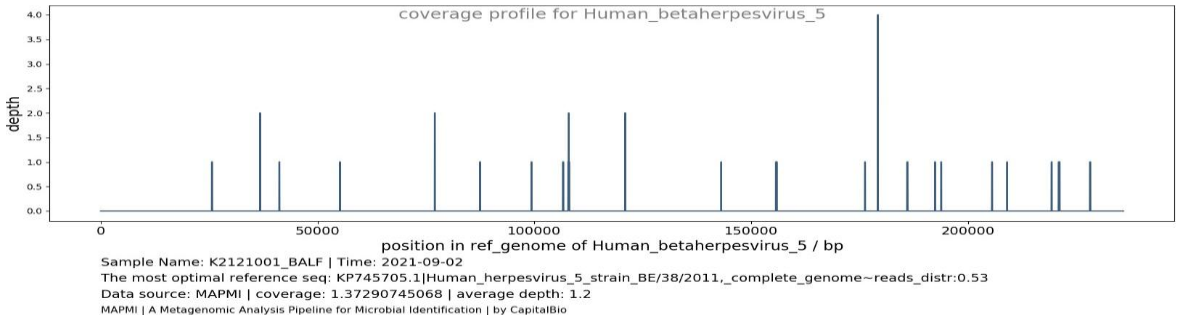

排名：2	种名：*Hu man betaherpesv iru s 7*	种分类号：10372

检出reads数：4　　覆盖度：0.24%

技术性注释
共有 4 条reads命中"10372 (*Hu man betaherpesv iru s 7*)"
命中的物种基因组总长度为 153080 bp，测到的该物种序列拼接后总长度为 373 bp，覆盖度为 0.24% # 测到的该物种序列的总碱基数为 519 bp，测到的该物种序列拼接后总长度为 373 bp，平均深度为 1.4X

命中参考序列：Human_betaherpesvirus_7

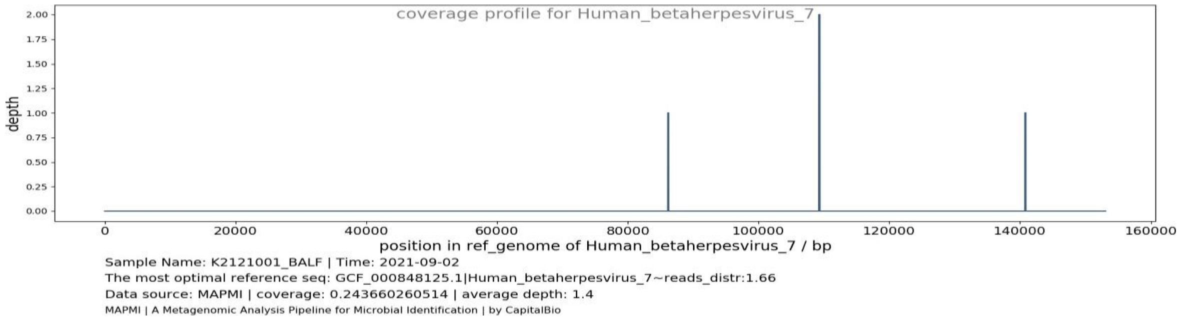

（三）阳性真菌、寄生虫详细技术信息

未检测到相关真菌、寄生虫。

（四）阳性结核分枝杆菌复合群详细技术信息

未检测到相关结核分枝杆菌复合群。

（五）阳性非结核分枝杆菌详细技术信息

未检测到相关非结核分枝杆菌。

（六）阳性支原体/衣原体/立克次氏体详细技术信息

未检测到相关支原体/衣原体/立克次氏体。

（七）阳性耐药基因详细技术信息

排名：1	种名: *ErmB [Enterococcu s faeciu m]*	种分类号：AR4985388

检出reads数：1241 覆盖度：99.2%

技术性注释
共有 1241 条reads命中“AR4985388（*ErmB [Enterococcu s faeciu m]*”
命中的物种基因组总长度为 749 bp，测到的该物种序列拼接后总长度为 745 bp，覆盖度为 99.2%
测到的该物种序列的总碱基数为 178123 bp，测到的该物种序列拼接后总长度为 745 bp，平均深度为 239.1X

命中参考序列：ErmB_[Enterococcus_faecium]

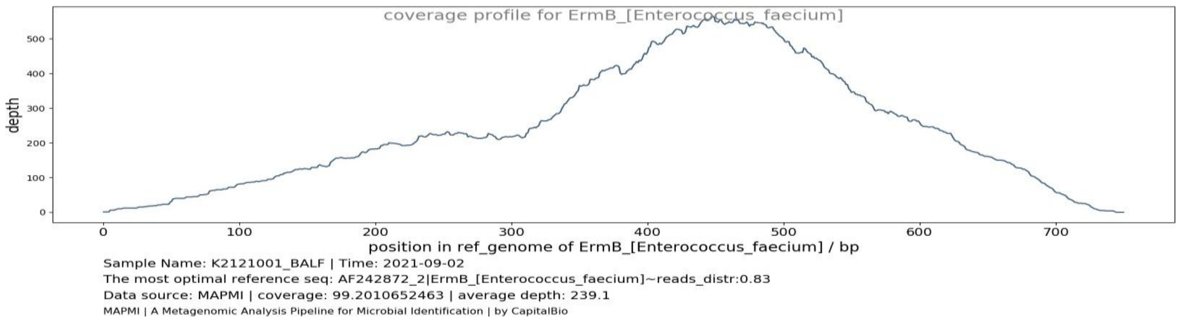

排名：2	种名: *efmA [Enterococcu s faeciu m]*	种分类号：AR4988967

检出reads数：364 覆盖度：99.77%

技术性注释
共有 364 条reads命中“AR4988967（*efmA [Enterococcu s faeciu m]*”
命中的物种基因组总长度为 1288 bp，测到的该物种序列拼接后总长度为 1287 bp，覆盖度为 99.77%
测到的该物种序列的总碱基数为 51917 bp，测到的该物种序列拼接后总长度为 1287 bp，平均深度为 40.3X

命中参考序列：efmA_[Enterococcus_faecium]

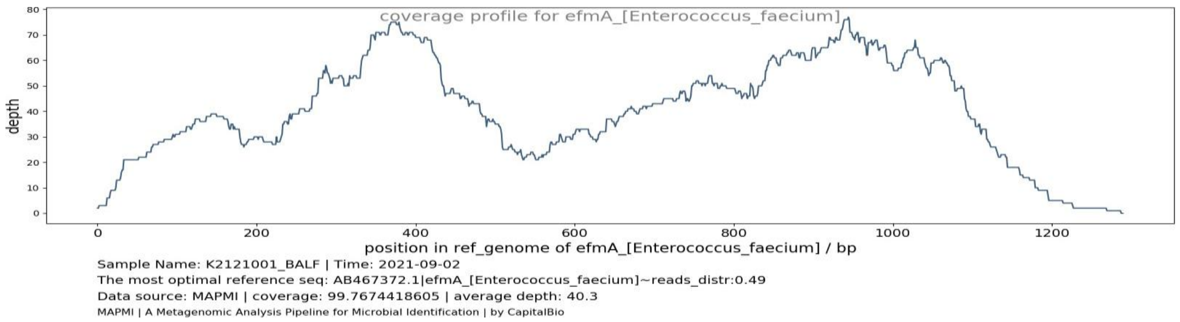

排名：3	种名: *AAC(6')-Ii [Enterococcu s faeciu m]*	种分类号：AR4987569
检出reads数：110 ｜ 覆盖度：98.73%		

\# 技术性注释 #
\# 共有 110 条reads命中“AR4987569（*AAC(6')-Ii [Enterococcu s faeciu m]*）”
\# 命中的物种基因组总长度为 550 bp，测到的该物种序列拼接后总长度为 545 bp，覆盖度为 98.73%
\# 测到的该物种序列的总碱基数为 15188 bp，测到的该物种序列拼接后总长度为 545 bp，平均深度为 27.9X

命中参考序列：AAC(6')-Ii_[Enterococcus_faecium]

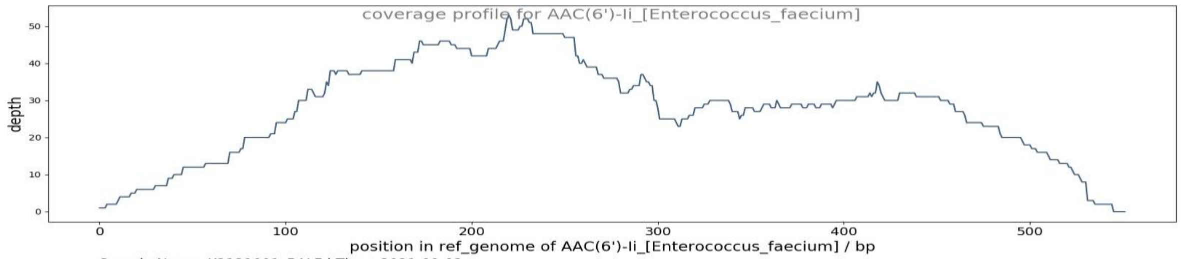

Sample Name: K2121001_BALF | Time: 2021-09-02
The most optimal reference seq: L12710|AAC(6')-Ii_[Enterococcus_faecium]~reads_distr:0.60
Data source: MAPMI | coverage: 98.731884058 | average depth: 27.9
MAPMI | A Metagenomic Analysis Pipeline for Microbial Identification | by CapitalBio

附录二-[疑似病原检测结果列表]

类型	属			种		
	中文名	拉丁文名	序列数	中文名	拉丁文名	序列数
G-	-	*Bru cella*	16	犬布鲁氏菌	*Bru cella ceti*	1
病毒	未检出					
真菌	未检出					
寄生虫	未检出					

附录三-[疑似定植菌检测结果列表]

类型	属			种			来源
	中文名	拉丁文名	序列数	中文名	拉丁文名	序列数	
G+	-	*Cory nebacteriu m*	15754	棒状杆菌属细菌	*Cory nebacteriu m simu lans*	188	呼吸道
G+	-	*Trophery ma*	398	惠普尔养障体	*Trophery ma w hipplei*	383	呼吸道
G+	-	*Actinomy ces*	112	衣氏放线菌	*Actinomy ces israelii*	74	口腔
病毒	未检出						
真菌	未检出						
寄生虫	未检出						
