# Supplementary material for: Tropheryma whipplei pneumonia: a retrospective case series of nine patients with treatment response
Source: Front Med (Lausanne). 2026 Jun 29;13:1883057. doi: 10.3389/fmed.2026.1883057 (PMC13357807; doi:10.3389/fmed.2026.1883057)
Supplement: Supplementary file 5 [file Data_Sheet_5.PDF]

一、样本与诊断信息

受检者基本信息

姓名：

性别：女

年龄：65

电话：—

住院号：—

床号：—

受检者临床诊断信息

临床症状：—

临床诊断：肺部感染

血常规：

WBC（10^9/L）	淋巴细胞（%）	中性粒细胞（%）	CRP（mg/L）	PCT（ng/ml）
—	—	—	—	—

其他结果：—

重点关注：细菌，真菌，支/衣原体

近期用药：—

样本信息

条码号：HZ232S0105921

样本编号：HZXD2307784BA

样本类型：肺泡灌洗液

样本体积：5ml

样本采集时间：2023-09-30 00:00:00

样本接收时间：2023-09-30 23:41:00

样本质量：质检合格

送检医生：林医生

送检科室：RICU

送检单位：福建省立医院

检测项目：Plseq®DNA病原检测产品

二、检测结果

检出指标	序列数(RPTM)[1]	阳性参考范围	定性结果[2]
蜡样芽孢杆菌群	155	≥20	细菌阳性
Dermacoccus abyssi（皮生球菌属细菌）	126	≥20	细菌阳性
*屎肠球菌	54	≥20	细菌阳性
*近平滑念珠菌	353	≥20	真菌阳性
球支孢霉	182	≥20	真菌阳性
*惠普尔养障体	18	≥20	细菌疑似
*铜绿假单胞菌	10	≥20	细菌疑似
龟分枝杆菌	1	≥20	细菌疑似

*标记说明可在中国肺炎网病原循证数据库检索到相关文献资料，本报告将展示相关资料（微生态除外）。无*标记说明中国肺炎网病原循证数据库未收录该微生物，本报告将不展示相关资料。

[1]RPTM，每一千万条测得序列包含的阳性序列条数(Reads per ten million)。

[2]定性结果同时也参考了内置的置信度指数计算模型值，用于描述检出物种的技术可靠度，主要包括阳性、疑似、人体微生态等结果。

本报告内容仅供专业的研究人员及临床医生参考，不作为临床确诊的唯一依据。

检验人：

审核人：

报告日期：2023-10-02 09:00
