# Supplementary material for: Tropheryma whipplei pneumonia: a retrospective case series of nine patients with treatment response
Source: Front Med (Lausanne). 2026 Jun 29;13:1883057. doi: 10.3389/fmed.2026.1883057 (PMC13357807; doi:10.3389/fmed.2026.1883057)
Supplement: Supplementary file 6 [file Data_Sheet_6.PDF]

微岩医学
WILLINGMED

◎ 让病原无处可藏

PIseq® DNA
病原宏基因组检测报告

送检单位：福建省立医院
送检科室：RICU
住 院 号：—
床 号：—

患者姓名：
条 码 号：HZ232S0105921
样本类型：肺泡灌洗液
报告日期：2023-10-02 09:00

三、附表 - 详细检测信息

1、样本中物种核酸丰度比例

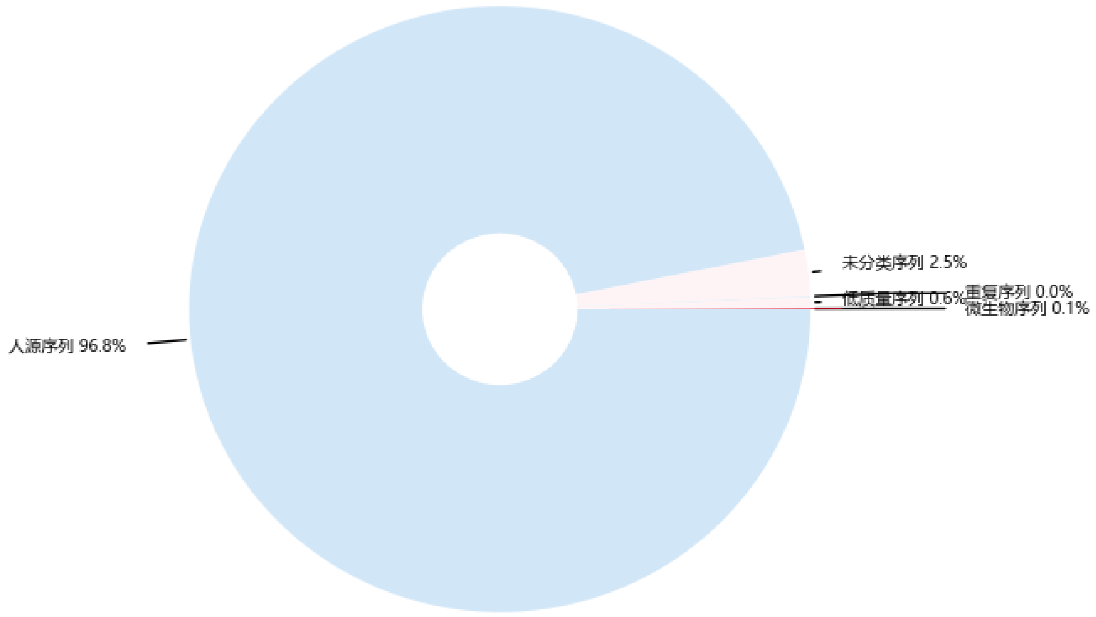

2、病毒筛查结果

序号	属信息		种信息			
	属名	序列数	种名	序列数	覆盖度	相对丰度
	未检出					

3、细菌筛查结果

序号	属信息		种信息			
	属名	序列数	种名	序列数	覆盖度	相对丰度

<table>
<tr><td rowspan="3">1</td><td>Bacillus
（芽孢杆菌属）</td><td>159</td><td>Bacillus_cereus_group
（蜡样芽孢杆菌群）</td><td>155</td><td>7361 bp
0.14%</td><td>2.6%</td></tr>
<tr><td colspan="6">蜡样芽孢杆菌群包含炭疽芽孢杆菌、蜡样芽孢杆菌、蕈状芽孢杆菌、假蕈状芽孢杆菌、苏云金芽孢杆菌和韦氏芽孢杆菌，革兰氏阳性细菌兼性厌氧菌。该菌属细菌广泛存在于土壤、动物肠道、植物体内、空气以及水体等环境中。临床上，蜡样芽胞杆菌群的细菌是条件致病菌，其引起的局部感染（ 如眼、皮肤、伤口 ） 和系统感染（ 如菌血症、脓毒症、脑膜炎、腹膜炎、心内膜炎、呼吸道及泌尿道感染 ）， 系统感染最常见于有基础疾病（如癌症和糖尿病 ）导致免疫功能受损的患者。</td></tr>
<tr><td colspan="6">略</td></tr>
<tr><td rowspan="3">2</td><td>Dermacoccus
（皮生球菌属）</td><td>136</td><td>Dermacoccus_abyssi
（Dermacoccus abyssi（皮生球菌属细菌））</td><td>126</td><td>15870 bp
0.53%</td><td>2.1%</td></tr>
<tr><td colspan="6">Dermacoccus abyssi是一种革兰氏阳性球菌，为皮生球菌属细菌，广泛分布于自然界中，在空气、水、食品和居室等外环境中。但暂未调研到该菌相关临床意义的文献，其具体临床意义需临床医生根据患者的临床症状进一步判断。</td></tr>
<tr><td colspan="6">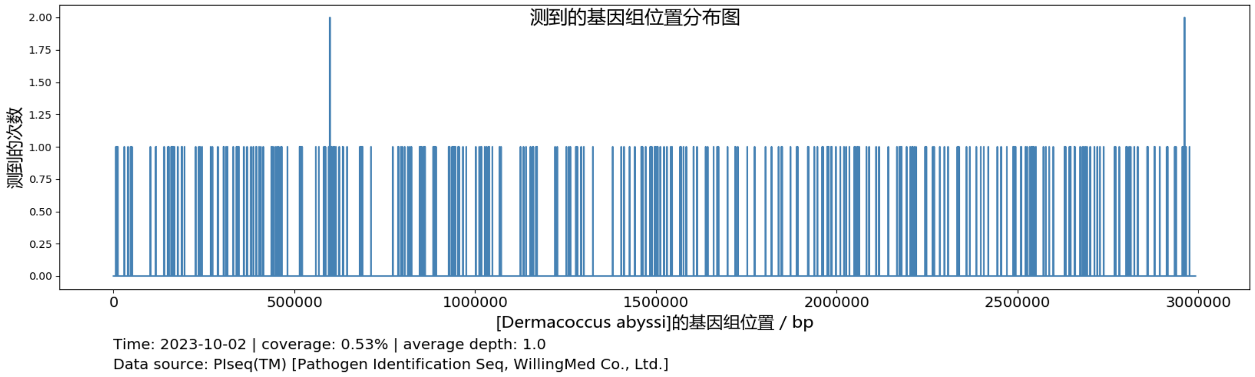
</td></tr>
<tr><td rowspan="3">3</td><td>Enterococcus
（肠球菌属）</td><td>85</td><td>Enterococcus_faecium
（屎肠球菌）</td><td>54</td><td>8898 bp
0.32%</td><td>0.9%</td></tr>
<tr><td colspan="6">屎肠球菌是一种革兰氏阳性兼性厌氧球菌，为肠球菌属细菌。该菌属细菌广泛分布于大自然中如泥土、植物、水、食物和动物中，也可定植于人体的胃肠道，一般其他部位存在（如泌尿生殖道、口腔、皮肤，特别是会阴部）相对较少。该菌是条件致病菌，能够在人体中引起多种感染，许多感染被认为是从胃肠道中主要居住位置迁移而引起的，通常主要感染尿道、血液、心内膜、烧伤和手术伤口、腹部、胆道、导尿管，也引起其他与植入性医疗设备相关的感染。经调研提示该菌有青霉素、氨苄西林、万古霉素、庆大霉素、链霉素等抗菌药物，其具体用药选择需临床医生根据患者的临床症状进一步判断。</td></tr>
<tr><td colspan="6">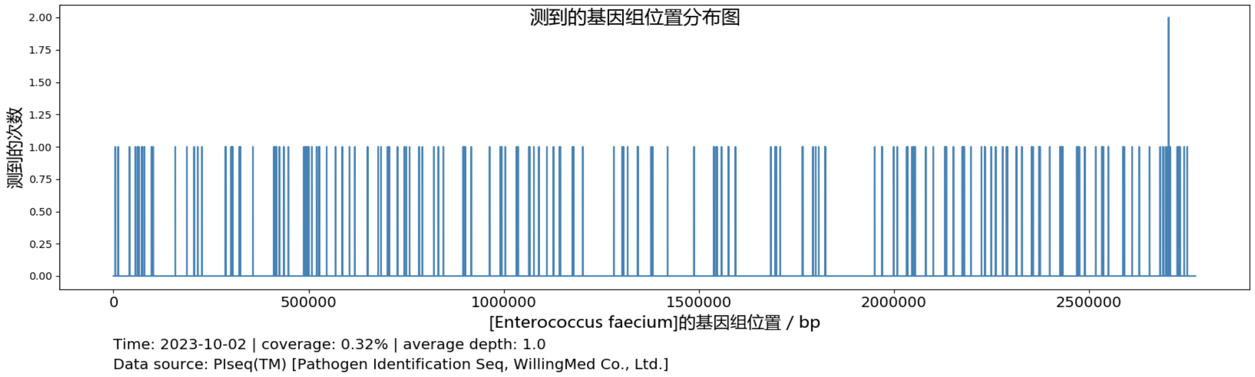
</td></tr>
</table>

<table>
<tr><td rowspan="3">4</td><td>Tropheryma
(养障体属)</td><td>18</td><td>Tropheryma_whipplei
(惠普尔养障体)</td><td>18</td><td>2107 bp
0.23%</td><td>0.3%</td></tr>
<tr><td colspan="6">惠普尔养障体是一种革兰氏阳性苛养杆菌，为养障体属细菌，可存在于各种环境中如土壤、污水，也可从健康人唾液、龈下菌斑、肠道活检、粪便中分离出。该菌为条件致病菌，主要通过家庭成员之间的口腔、粪-口传播、水滴或空气传播等方式感染，主要引起惠普尔氏病，惠普尔病临床表现多种多样，无特异性，包括慢性腹泻、吸收不良、游走性关节炎、体重减轻、淋巴结肿大、葡萄膜炎/视网膜炎/眼内炎、心内膜炎、脑炎、免疫重建炎性综合征、急性肠胃炎、胸腔积液、肺浸润、纵隔淋巴结病等。经调研提示青霉素、头孢曲松、复方磺胺甲噁唑、类固醇等抗菌药物可用于惠普尔养障体感染治疗，其具体用药选择需临床医生根据患者的临床症状进一步判断。</td></tr>
<tr><td colspan="6">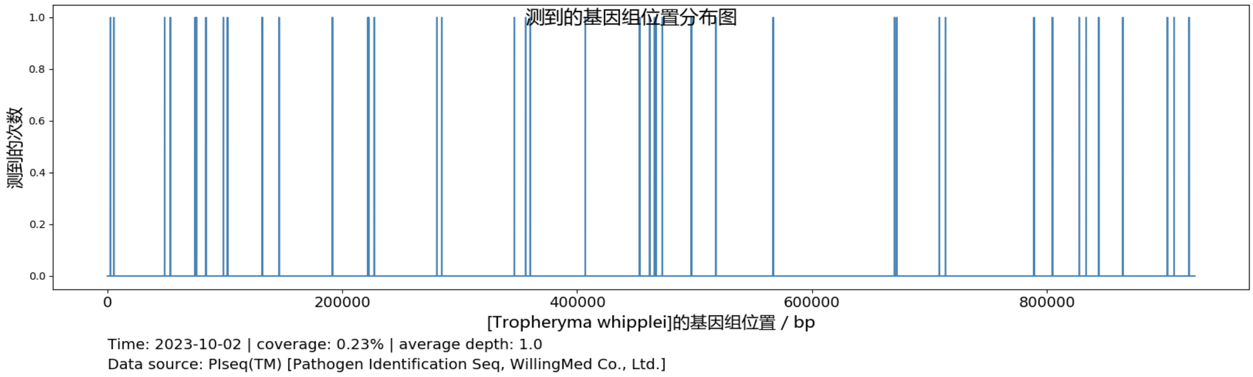
</td></tr>
<tr><td rowspan="3">5</td><td>Pseudomonas
(假单胞菌属)</td><td>16</td><td>Pseudomonas_aeruginosa
(铜绿假单胞菌)</td><td>10</td><td>1225 bp
0.02%</td><td>0.17%</td></tr>
<tr><td colspan="6">铜绿假单胞菌原称绿脓杆菌，是一种无芽孢革兰氏阴性需氧杆菌，该菌广泛分布在潮湿的环境中，如土壤、植物、蔬菜、自来水和台面等，也存在于正常人的皮肤、呼吸道和肠道内。该菌是医院内感染的主要病原菌之一，患代谢性疾病、血液病和恶性肿瘤的患者，以及术后或某些治疗后的患者易感染本菌，该菌可引起身体各个部位的感染，主要为肺部感染，也有引起菌血症、心内膜炎、肺炎、骨感染、恶性外耳炎、中枢神经系统感染、眼部感染（角膜炎/溃疡眼内炎）、气道慢性感染、皮肤软组织感染、尿路感染、艾滋病患者的感染、发热伴中性粒细胞减少综合症患者的感染等，经调研提示该菌有头孢吡肟、头孢他啶、妥布霉素、美罗培南、环丙沙星等抗菌药物，其具体用药选择需临床医生根据患者的临床症状进一步判断。</td></tr>
<tr><td colspan="6">略</td></tr>
<tr><td rowspan="3">6</td><td>Mycobacterium_chelonae_complex
(分枝杆菌属)</td><td>1</td><td>Mycobacteroides_chelonae
(龟分枝杆菌)</td><td>1</td><td>98 bp
0.00%</td><td>0.0083%</td></tr>
<tr><td colspan="6">龟分枝杆菌是一种革兰氏阳性杆菌，为龟-脓肿分枝杆菌复合群成员，可广泛分布于外界环境如含水分较多的地方（湖、河和土壤）和正常人及动物机体中，该菌是条件致病菌，可侵犯人体的肺脏、淋巴结、骨骼、关节、皮肤和软组织等组织器官，并可导致全身播散性疾病。经调研提示该菌有克拉霉素等抗菌药物，其具体用药选择需临床医生根据患者的临床症状进一步判断。</td></tr>
<tr><td colspan="6">略</td></tr>
</table>

4、真菌筛查结果

<table>
<tr><td rowspan="2">序号</td><td colspan="2">属信息</td><td colspan="4">种信息</td></tr>
<tr><td>属名</td><td>序列数</td><td>种名</td><td>序列数</td><td>覆盖度</td><td>相对丰度</td></tr>
</table>

<table>
<tr><td rowspan="3">1</td><td>Candida
(念珠菌属)</td><td>355</td><td>Candida_parapsilosis
(近平滑念珠菌)</td><td>353</td><td>41908 bp
0.32%</td><td>61.2%</td></tr>
<tr><td colspan="6">近平滑念珠菌是一种卵球形酵母菌，为念珠菌属真菌，可在自然界呈现广泛分布。不仅在植物、土壤、海水中可以分离到，还可在健康的人和其他哺乳动物的黏膜表面、皮肤以及指甲中发现。该菌是条件致病菌，一般低体重新生儿、免疫力低下人群，尤其是HIV感染者、器官移植后病人，以及腹腔手术患者等易感染本菌，可引起皮肤浅表感染、中耳炎、鼻窦炎、眼和泌尿生殖道感染。经调研提示该菌属真菌有氟康唑、两性霉素B、卡泊芬净、伊曲康唑等抗菌药物，其具体用药选择需临床医生根据患者的临床症状进一步判断。</td></tr>
<tr><td colspan="6">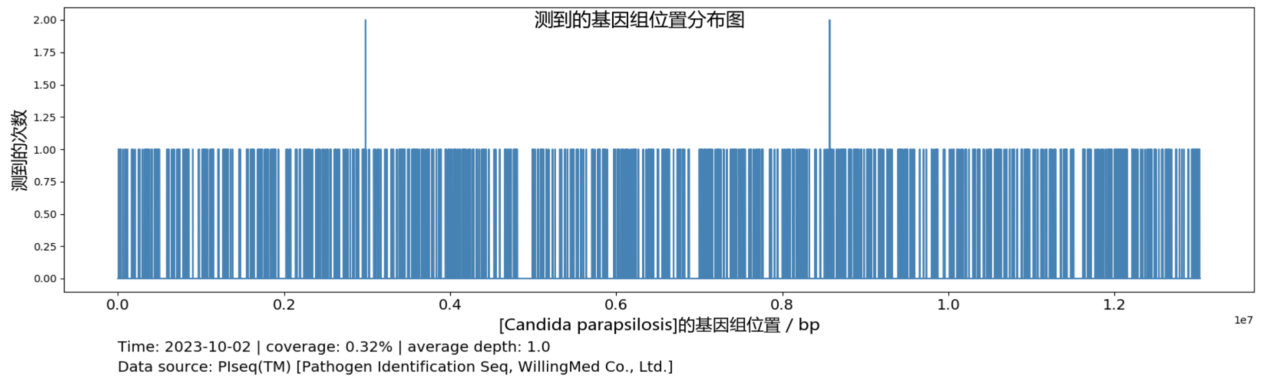
</td></tr>
<tr><td rowspan="3">2</td><td>Cladosporium
(枝孢霉属)</td><td>184</td><td>Cladosporium_sphaerospermum
(球支孢霉)</td><td>182</td><td>19599 bp
0.07%</td><td>31.6%</td></tr>
<tr><td colspan="6">球支孢霉是一种腐生丝状真菌，为枝孢霉属真菌，常广泛存在于自然界中。该菌是条件致病菌，主要通过空气传播，可存在于室内和室外空气中，不仅从住宅和植物中分离到，还可从人类中分离到，一般免疫力低下的老年人或者外伤等直接接触条件下易感本菌，可引起着色真菌病、脑膜炎，皮肤和皮下组织感染、肺部真菌感染等。经调研提示该菌有伊曲康唑、两性霉素B、酮康唑、制霉菌素等抗菌药物，其具体用药选择需临床医生根据患者的临床症状进一步判断。</td></tr>
<tr><td colspan="6">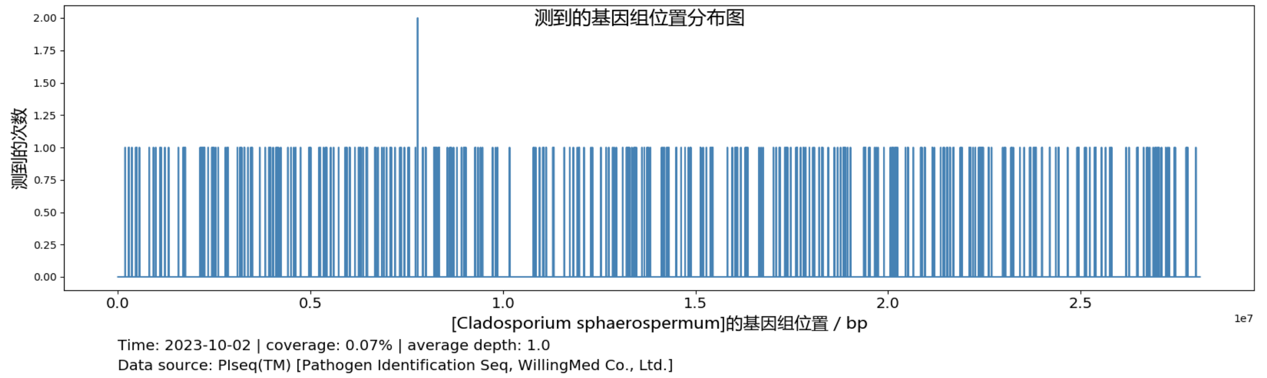
</td></tr>
</table>

5、寄生虫筛查结果

序号	属信息		种信息			
	属名	序列数	种名	序列数	覆盖度	相对丰度
	未检出					

6、耐药基因筛查结果

序号	检测出耐药基因	抗生素类别	可能对应物种

未检出

7、毒力基因筛查结果

序号	毒力因子	基因名称	功能	可能对应物种
	未检出			

8、检出常见人体微生态细菌列表

序号	属信息		种信息				注释
	属名	序列数	种名	序列数	覆盖度	相对丰度	
	未检出						

9、中国肺炎网病原循证数据库

中国肺炎研究
CAP-China

二代测序（next generation sequencing，NGS）因其高敏感性在呼吸道病原诊断中得到广泛应用。呼吸道感染临床表现多样，病原体种类复杂，常见一个标本中检出多种甚至十余种微生物，给临床解读带来了巨大挑战，影响了病原学诊断的临床实践。此时，临床医生经常需要花费大量时间查询相关文献，才有可能对检出微生物的临床意义做出正确判断。这不仅难以在短时间内进行系统和精准的查询，而且浪费了宝贵的时间。

为此，国家呼吸医学中心、中国肺炎研究网、中日友好医院研究团队开发了CAP-China病原检索工具。该工具借助人工神经网络算法筛选出30万余篇呼吸道病原致病文献，通过提取关键信息，包括标本类型、研究例数、免疫状态、临床症状等，形成了呼吸道病原循证数据库，为临床医生提供高效快捷的病原检索服务。（详情请登录中国肺炎研究网：https://www.chinapneumonia.cn/pathogens）。

微岩医学将中国肺炎研究网（CAP-China）呼吸道病原循证数据库整合进每一份mNGS报告中，病原循证数据库可显示该微生物在不同标本部位被报道的频次、以及在不同免疫状态患者中被报道的频次，从而帮助临床快速系统判断该微生物的临床意义及可能成为责任致病原的概率。为进一步友好、可视化显示，微岩医学基于中国肺炎网病原循证数据库的大数据信息，借助神经网络Bert模型，参考国内外公认权威信息（如WHO《真菌优先病原体列表（FPPL）》、中国《人间传染的病原微生物目录》、中国《生物安全等级目录》等），计算了上千种微生物在呼吸道标本检出时的临床致病概率。如下图示例所示：

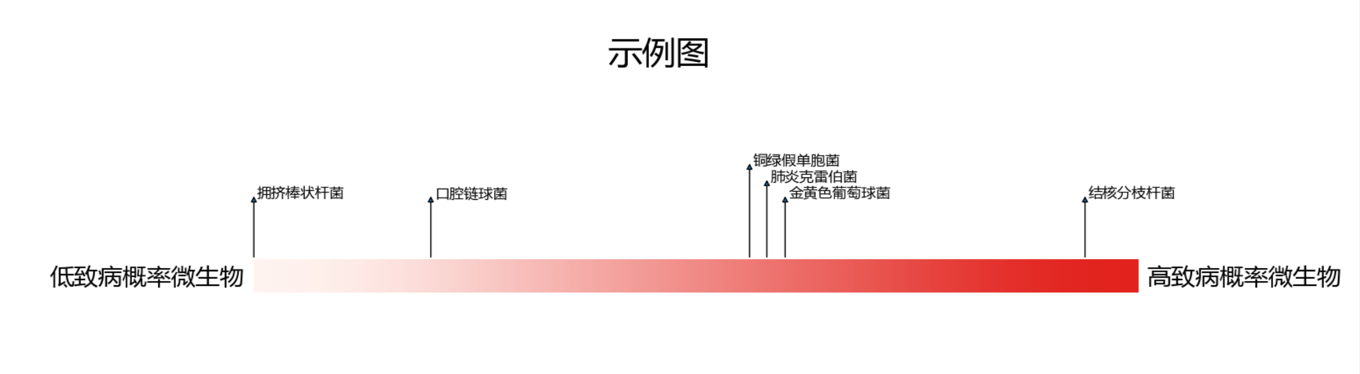

本报告检出微生物呼吸道致病概率汇总图

本标本中检出微生物的临床致病概率如下图所示：

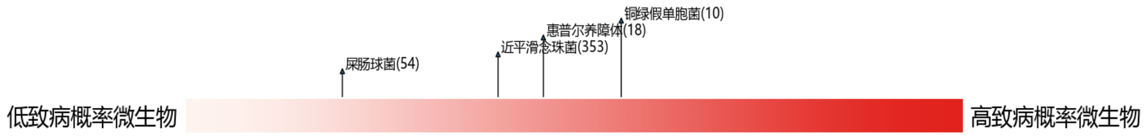

备注：

1.本报告微生物肺部感染临床致病概率一览表如上图所示，如检出微生物靠近左侧，通常意味着其临床致病概率较低，例如拥挤棒状杆菌等，但在某些情况下，如脓肿患者、吸入性肺炎患者，该类微生物的临床致病能力仍不容忽视；如检出微生物靠近右侧，则通常意味着其临床致病概率较高，例如结核分枝杆菌等。

2.该致病概率图中结果仅适于呼吸道标本如痰液、肺泡灌洗支气管灌洗液等，不适用于其他无菌标本。

3.微生物临床致病概率仅用于科研，可为临床提供一定程度参考，其不是临床感染诊断的唯一依据，请结合其他检测结果及临床实际情况共同作出临床诊断。

4.括号内为该微生物在本标本中检出的归一化reads数。

中国肺炎网病原循证数据库检索信息						
病原名称	免疫状态	呼吸道常见标本	无菌体液	肺组织	未分类标本	总量
屎肠球菌（54）	免疫正常文献篇数	3	13	2	20	38
	免疫低下文献篇数	0	9	1	8	18
	总数量	3	22	3	28	56

本报告的标本类型为**肺泡灌洗液**，属于**呼吸道常见标本**，该微生物在中国肺炎网病原循证数据库检索到临床致病相关文献共**56**篇，其中来自免疫正常患者的文献总数为**38**篇，免疫低下患者的文献总数为**18**篇（占比为**32%**）。

屎肠球菌在**呼吸道标本**的临床致病概率经神经网络Bert模型计算结果如下图所示：

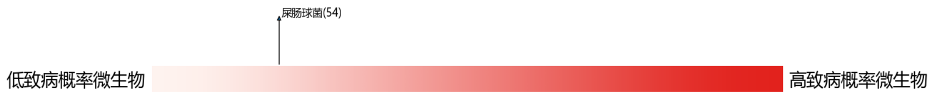

部分文献检索结果如下:

1.Co-Infections in Critically Ill Patients with or without COVID-19: A Comparison of Clinical Microbial Culture Findings. International journal of environmental research and public health, 2021, [IF=4.614], [样本量：none]
https://pubmed.ncbi.nlm.nih.gov/33923992/

2.Fatal strongyloidiasis after corticosteroid therapy for presumed chronic obstructive pulmonary disease. none, 2018, [IF=none], [样本量：1]
https://pubmed.ncbi.nlm.nih.gov/30425838/

3.[Experience of the selection and maintainence for donor lung: 62 cases attached]. none, 2017, [IF=none], [样本量：11-100]
https://pubmed.ncbi.nlm.nih.gov/27916031/

其它检索结果详见中国肺炎研究网

近平滑念珠菌（353）

免疫状态	呼吸道常见标本	无菌体液	肺组织	未分类标本	总量
免疫正常文献篇数	5	23	1	24	53
免疫低下文献篇数	4	12	0	10	26
总数量	9	35	1	34	79

本报告的标本类型为**肺泡灌洗液**，属于**呼吸道常见标本**，该微生物在中国肺炎网病原循证数据库检索到临床致病相关文献共**79**篇，其中来自免疫正常患者的文献总数为**53**篇，免疫低下患者的文献总数为**26**篇（占比为**33%**）。

近平滑念珠菌在**呼吸道标本**的临床致病概率经神经网络Bert模型计算结果如下图所示：

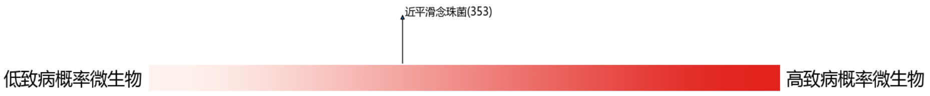

部分文献检索结果如下:

1.Co-Infections in Critically Ill Patients with or without COVID-19: A Comparison of Clinical Microbial Culture Findings. International journal of environmental research and public health, 2021, [IF=4.614], [样本量：none]
https://pubmed.ncbi.nlm.nih.gov/33923992/

2.Rapid detection of Candida species in bronchoalveolar lavage fluid from patients with pulmonary symptoms. none, 2016, [IF=none], [样本量：11-100]
https://pubmed.ncbi.nlm.nih.gov/26887241/

3.Enterococcus faecium Mediastinitis Complicated by Disseminated Candida parapsilosis Infection after Congenital Heart Surgery in a 4-Week-Old Baby. none, 2015, [IF=none], [样本量：1]
https://pubmed.ncbi.nlm.nih.gov/26605096/

其它检索结果详见中国肺炎研究网

惠普尔养障体（18）

免疫状态	呼吸道常见标本	无菌体液	肺组织	未分类标本	总量
免疫正常文献篇数	2	4	1	5	12
免疫低下文献篇数	2	0	1	3	6
总数量	4	4	2	8	18

本报告的标本类型为**肺泡灌洗液**，属于**呼吸道常见标本**，该微生物在中国肺炎网病原循证数据库检索到临床致病相关文献共**18**篇，其中来自免疫正常患者的文献总数为**12**篇，免疫低下患者的文献总数为**6**篇（占比为**33%**）。

惠普尔养障体在**呼吸道标本**的临床致病概率经神经网络Bert模型计算结果如下图所示：

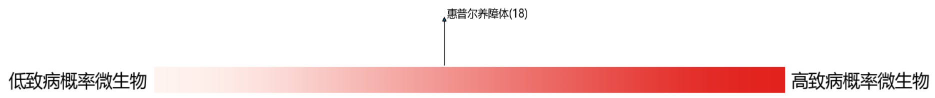

部分文献检索结果如下:

1.Severe pneumonia in adults caused by Tropheryma whipplei and Candida sp. infection: a 2019 case series. BMC pulmonary medicine, 2021, [IF=3.32], [样本量：11-100]
https://pubmed.ncbi.nlm.nih.gov/33451316/

2.Acute infections caused by Tropheryma whipplei. Future microbiology, 2017, [IF=3.553], [样本量：11-100]
https://pubmed.ncbi.nlm.nih.gov/28262041/

3.Pulmonary coinfection of Mycobacterium tuberculosis and Tropheryma whipplei: a case report. none, 2021, [IF=none], [样本量：none]
https://pubmed.ncbi.nlm.nih.gov/34243811/

4.Pulmonary parenchymal involvement caused by Tropheryma whipplei . none, 2021, [IF=none], [样本量：1]
https://pubmed.ncbi.nlm.nih.gov/34131590/

5.Tropheryma whipplei DNA in bronchoalveolar lavage samples: a case control study. none, 2017, [IF=none], [样本量：none]
https://pubmed.ncbi.nlm.nih.gov/27432769/

其它检索结果详见中国肺炎研究网

铜绿假单胞菌（10）

免疫状态	呼吸道常见标本	无菌体液	肺组织	未分类标本	总量
免疫正常文献篇数	506	496	48	1488	2538
免疫低下文献篇数	161	189	37	452	839
总数量	667	685	85	1940	3377

本报告的标本类型为**肺泡灌洗液**，属于**呼吸道常见标本**，该微生物在中国肺炎网病原循证数据库检索到临床致病相关文献共**3377**篇，其中来自免疫正常患者的文献总数为**2538**篇，免疫低下患者的文献总数为**839**篇（占比为**25%**）。

铜绿假单胞菌在**呼吸道标本**的临床致病概率经神经网络Bert模型计算结果如下图所示：

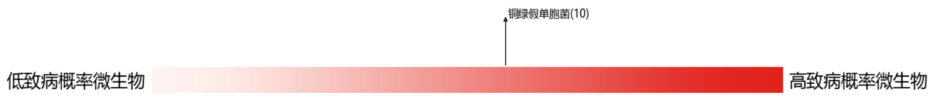

部分文献检索结果如下:

1.Etiology of Severe Community-Acquired Pneumonia in Adults Based on Metagenomic Next-Generation Sequencing: A Prospective Multicenter Study. Infectious diseases and therapy, 2020, [IF=6.119], [样本量：>100]
https://pubmed.ncbi.nlm.nih.gov/33170499/

2.Antibiotic resistance heterogeneity and LasR diversity within Pseudomonas aeruginosa populations from pneumonia in intensive care unit patients. International journal of antimicrobial agents, 2021, [IF=15.441], [样本量：11-100]
https://pubmed.ncbi.nlm.nih.gov/33857540/

3.Time to Result for Pathogen Identification and Antimicrobial Susceptibility Testing of Bronchoalveolar Lavage and Endotracheal Aspirate Specimens in U.S. Acute Care Hospitals. Journal of clinical microbiology, 2021, [IF=11.677], [样本量：>100]
https://pubmed.ncbi.nlm.nih.gov/32878953/

其它检索结果详见中国肺炎研究网

注：
1.所有文献来源CAP-China（中国肺炎研究网）https://www.chinapneumonia.cn/pathogens。
2.中国肺炎网病原循证数据库检索信息表中呈现的是设置不同条件时，自CAP -China检索到某病原临床致病相关文献的篇数。
3.文献按照物种-标本类型-时间-影响因子的顺序规则优先展示。
4.文献搜索数目和文献报道的病原研究结果，不能直接作为最终临床诊断依据；临床诊断需结合患者临床表征、影像学改变和宿主特点等综合判断。
5.呼吸道常见标本包含：肺泡灌洗液、痰、气管吸出物等；无菌体液包含：血液、脑脊液、胸腹水、尿液等；肺组织样本包括支气管镜活检组织、无菌环境获取的肺组织(包含手术组织、经皮穿刺组织等)；未分类指未归入现有等级的标本类型。
6.联系邮箱：cap-china2015@vip.163.com

四、质量控制信息

指标	结果
阳性对照	合格
阴性对照	合格
内对照	合格
GC%	42%
Q30	93.907%

测序质量分布图：

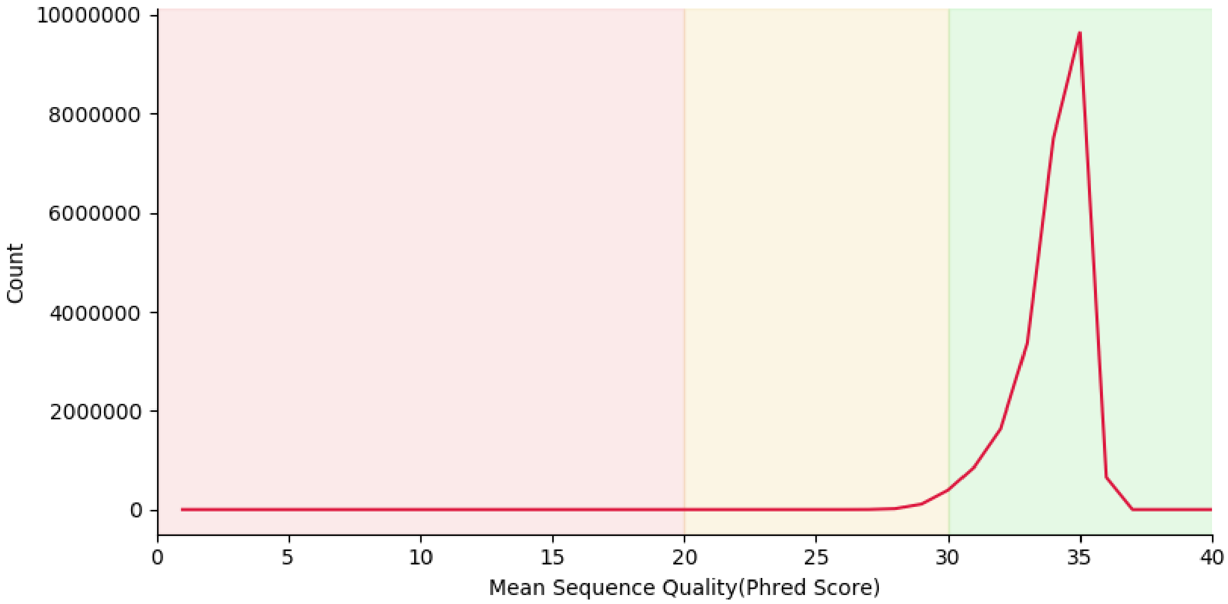

注：以上图表说明此次检测测序数据质量合格，结果可信。

五、检验原理和术语、名词说明

PIseq®（Pathogen Identification Sequencing）是微岩医学基于宏基因组学二代测序技术（mNGS，metagenomics next-generation sequencing）开发的病原微生物检测技术，该技术无需预判感染微生物，直接对临床样本中的遗传物质（核酸）进行全面无偏的检测。PIseq®技术可覆盖25000多种病原体，包括11836种细菌、11021种病毒、1872种真菌、421种寄生虫、153种分枝杆菌、118种支原体/衣原体、以及105种立克次体。本检测通过高效病原富集核酸提取技术，结合微岩自建的微生物基因标签序列数据库及中国流行病原知识库，采用独有的双引擎物种鉴定算法，全面扫描样本中存在的病原微生物、耐药基因和毒性元件等信息，并提供大信息量的检测报告及报告解读， 在不明感染、疑难危重及免疫缺陷感染患者中有很高的临床应用价值。

- 检测流程

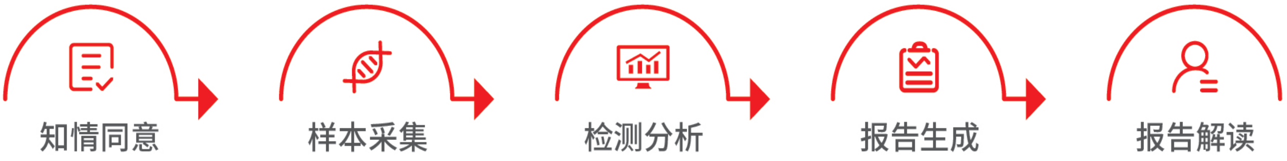

- 检测范围

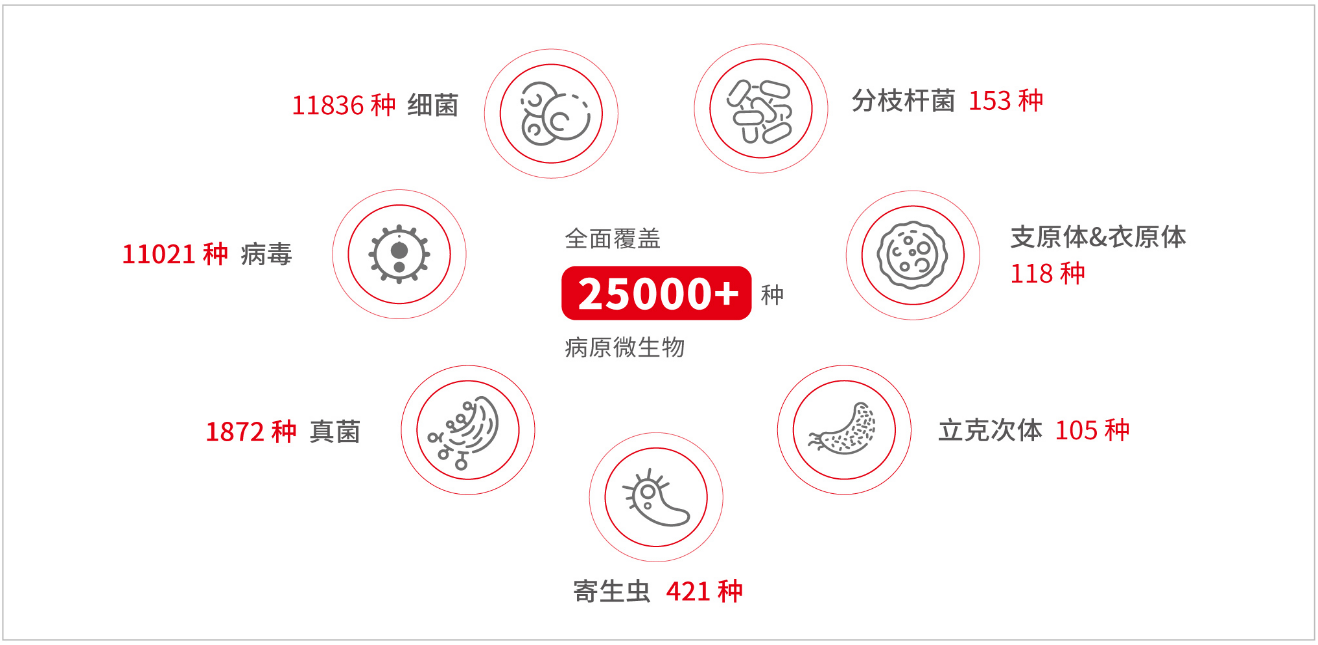

术语和名词说明

◊ **宏基因组学技术**：即基于宏基因组学的二代测序技术，又称mNGS技术（metagenomics next-generation sequencing），是将样本中的基因组核酸随机打断后利用二代测序测定所有核酸序列的技术。

◊ **疑似物种**：指不太确定样本中是否存在检出的物种，检测指标处于技术的检测灰区，结果仅供临床参考。

序列数：指在属/种水平上检出的该微生物的特异性序列数目。

◊ **覆盖度**：指检出的该微生物所有序列经向基因组回拼后，能够覆盖该物种基因组的百分比。

◊ **相对丰度**：指除去宿主序列之后，某微生物物种序列在相应大类物种（通常分成细菌、真菌、病毒、寄生虫4大类）中的分布比例。

◊ **覆盖谱图**：指以微生物全基因组碱基位置为横坐标，该位置被测到次数为纵坐标的二维图表，该图表描述了随机测序测到的基因组上的所有位置，通常，被测到的位置越多、越平均，则表明微生物真实存在的概率越高。

◊ **耐药基因**：指可能会导致细菌产生耐药性的基因序列。

◊ **毒力基因**：指可能会导致细菌产生毒力的基因序列。

◊ **人体微生态**：指人体内的微生物生态群落，主要指分布于人体的消化道、上呼吸道、皮肤、尿道、肠道等与人体共生的微生物，在一定条件下可能成为致病菌。

◊ **碱基质量分布图**：高通量测序每测完一个碱基，会给出一个相应的测序质量值，用于衡量测序仪的准确度，称为Q_{phred}(Phred quality score)，计算公式为$Q_{phred} = -10log10P_{error}$，因而若图中所示纵坐标数值为30，则代表该处碱基的测序错误概率为1/1000。

◊ **GC%**：指测到的所有序列的平均GC碱基百分含量。

六、参考文献、名词说明

[1]. Wilson MR, Naccache SN, Samayoa E, et al. Actionable diagnosis of neuroleptospirosis by next-generation sequencing. N Engl J Med. 2014;370(25):2408-2417.

[2]. Blauwkamp TA, Thair S, Rosen MJ, et al. Analytical and clinical validation of a microbial cell-free DNA sequencing test for infectious disease. Nat Microbiol. 2019;4(4):663-674.

[3]. Gu W, Deng X, Lee M, et al. Rapid pathogen detection by metagenomic next-generation sequencing of infected body fluids. Nat Med. 2021;27(1):115-124.

[4]. Charalampous T, Kay GL, Richardson H, et al. Nanopore metagenomics enables rapid clinical diagnosis of bacterial lower respiratory infection. Nat Biotechnol. 2019;37(7):783-792.

[5]. Liu D, Zhou H, Xu T, et al. Multicenter assessment of shotgun metagenomics for pathogen detection. EBioMedicine. 2021;74:103649.

[6]. Chen H, Zheng Y, Zhang X, et al. Clinical evaluation of cell-free and cellular metagenomic next-generation sequencing of infected body fluids. J Adv Res. 2023:S2090-1232(23)00068-1

[7]. Gu W, Miller S, Chiu CY. Clinical Metagenomic Next-Generation Sequencing for Pathogen Detection. Annu Rev Pathol. 2019;14:319-338.

[8]. Chiu CY, Miller SA. Clinical metagenomics. Nat Rev Genet. 2019;20(6):341-355.

[9]. Han D, Li R, Shi J, Tan P, Zhang R, Li J. Liquid biopsy for infectious diseases: a focus on microbial cell-free DNA sequencing. Theranostics. 2020;10(12):5501-5513.

[10]. 中华医学会检验医学分会临床微生物学组, 中华医学会微生物学与免疫学分会临床微生物学组, 中国医疗保健国际交流促进会临床微生物与感染分会. 宏基因组高通量测序技术应用于感染性疾病病原检测中国专家共识 [J] . 中华检验医学杂志, 2021, 44(2) : 107-120.

[11]. 中华医学会检验医学分会. 宏基因组测序病原微生物检测生物信息学分析规范化管理专家共识 [J] . 中华检验医学杂志, 2021, 44(9) : 799-807.

[12]. 中华医学会呼吸病学分会. 下呼吸道感染宏基因组二代测序报告临床解读路径专家共识 [J] . 中华结核和呼吸杂志, 2023, 46(4) : 322-335.

[13]. Neyton LPA, Langelier CR, Calfee CS. Metagenomic Sequencing in the ICU for Precision Diagnosis of Critical Infectious Illnesses. Crit Care. 2023;27(1):90.

[14]. Edgeworth JD. Respiratory metagenomics: route to routine service. Curr Opin Infect Dis. Published online February 2, 2023.

[15]. Qu C, Chen Y, Ouyang Y, et al. Metagenomics next-generation sequencing for the diagnosis of central nervous system infection: A systematic review and meta-analysis. Front Neurol. 2022;13:989280.

七、检测方法局限性和免责声明

1. 本报告检测结果仅供临床参考，不能用于最终诊断，需临床结合患者情况进行综合诊治。

2. 本报告检测结果只对本次受检样品负责，如有疑义请在收到报告之日起10个工作日内与我们联系。

3. 本检测方法具有其自身技术局限性和检测范围，最低检测限为100 copies/mL(病毒为1000copies/mL)。

4. 本报告所采用的mNGS宏基因组测序技术，原理是基于鸟枪法随机测序技术，对于reads数较少的灰区、疑似病原检出结果，存在多次检测结果不一致的可能；

5. 本公司采用的pathoXtract宿主核酸剔除技术能将病原丰度提高100-1000倍，大大提高检测灵敏度，但随样本类型不同和具体剔除效果可不同，存在数据产出量波动的可能；

6. 本报告检测结果为阴性，并不代表该样本中一定不存在病原微生物，导致该结果的原因包括但不限于：

① 被检病原体浓度低于本方法的最低检测限；

② 被检病原体基因组序列未在本方法数据库中收录。

7. 研究表明，耐药基因与毒力因子基因型与实际表型可能不完全一致，因此本报告中的耐药基因和毒力因子基因检测结果仅供参考，不能作为临床诊断或用药的唯一依据。

8. 本报告中检出的常见人体微生态细菌，一般为人体正常菌群，检出结果仅供参考，具体致病性需临床医生综合患者情况进行判断，检出人体微生态细菌的原因包括但不限于：

① 样本采集部位定植有人体微生态细菌，如皮肤、呼吸道、肠道等；

② 样本采集过程中受到病人身上人体微生态细菌的污染；

③ 样本采集过程中受到采集或分装人员身上人体微生态细菌的污染；

④ 可能为造成感染的真正病原体。

9. 本检测报告无检验人、审核人签字无效，涂改无效，内容缺损无效，无检测专用章无效。

10. 该实验性能特性由微岩医学实验室确认，未经过国家食品药品监督管理总局的批准。

11. 本公司保留对本报告检测结果的最终解释权。

微岩医学
WILLINGMED

PIseq® DNA
病原宏基因组检测报告

微岩医学科技(北京)有限公司
WillingMed Technology (Beijing) Co., Ltd.

地址:北京市经济技术开发区经海四路156号经海产业园A3栋2层
邮箱:willingmed@willingmed.com

WY02-202306，All rights reserved

让 病 原 无 处 可 藏
